# Patients’ perceptions with musculoskeletal disorders regarding their experience with healthcare providers and health services: an overview of reviews

**DOI:** 10.1186/s40945-020-00088-6

**Published:** 2020-09-24

**Authors:** Alan Chi-Lun-Chiao, Mohammed Chehata, Kenneth Broeker, Brendan Gates, Leila Ledbetter, Chad Cook, Malene Ahern, Daniel I. Rhon, Alessandra N. Garcia

**Affiliations:** 1 Duke Department of Orthopedic Surgery, Duke University Division of Physical Therapy, Durham, North Carolina USA; 2 Duke University Medical Center Library, Durham, North Carolina USA; 3 Duke Department of Orthopedic Surgery, Duke University Division of Physical Therapy, Duke Clinical Research Institute, Durham, North Carolina USA; 4 University of Wollongong, Australian Health Services Research Institute, Sydney, New South Wales Australia; 5 Center for the Intrepid, Brooke Army Medical Center, San Antonio, TX USA; 6 College of Pharmacy & Health Sciences, Physical Therapy Program, Lillington, North Carolina USA

**Keywords:** Musculoskeletal disorder, Patient experience, Healthcare, Systematic review

## Abstract

**Objectives:**

This overview of reviews aimed to identify (1) aspects of the patient experience when seeking care for musculoskeletal disorders from healthcare providers and the healthcare system, and (2) which mechanisms are used to measure aspects of the patient experience.

**Data sources:**

Four databases were searched from inception to December 20th, 2019.

**Review methods:**

Systematic or scoping reviews examining patient experience in seeking care for musculoskeletal from healthcare providers and the healthcare system were included. Independent authors screened and selected studies, extracted data, and assessed the methodological quality of the reviews. Patient experience concepts were compiled into five themes from a perspective of a) *relational* and b) *functional* aspects. A list of mechanisms used to capture the patient experience was also collected.

**Results:**

Thirty reviews were included (18 systematic and 12 scoping reviews). Relational aspects were reported in 29 reviews and functional aspects in 25 reviews. For relational aspects, the most prevalent themes were “information needs” (education and explanation on diseases, symptoms, and self-management strategies) and “understanding patient expectations” (respect and empathy). For functional aspects, the most prevalent themes were patient’s “physical and environmental needs,” (cleanliness, safety, and accessibility of clinics), and “trusted expertise,” (healthcare providers’ competence and clinical skills to provide holistic care). Interviews were the most frequent mechanism identified to collect patient experience.

**Conclusions:**

Measuring patient experience provides direct insights about the patient’s perspectives and may help to promote better patient-centered health services and increase the quality of care. Areas of improvement identified were interpersonal skills of healthcare providers and logistics of health delivery, which may lead to a more desirable patient-perceived experience and thus better overall healthcare outcomes.

**Trial registration:**

**Systematic review registration: PROSPERO** (CRD42019136500).

## Introduction

Musculoskeletal (MSK) disorders including neck and low back pain, hip and knee osteoarthritis, and rheumatoid arthritis, are some of the most burdensome conditions in terms of disability worldwide, associated high healthcare utilization and costs [1–3]. Because of the high incidence of chronicity [4] of these disorders, seeking treatment from and recurring visits to healthcare services are frequent and common [5–7]. Efforts to optimize the overall quality of healthcare could promote better outcomes and patient satisfaction as well as minimize the burden of healthcare delivery in MSK settings [8–10]. Patient experience has been recognized as a significant contributing factor to the quality of healthcare and has recently drawn more research interest [5, 11–14]. A deeper understanding of patients’ experience of healthcare-seeking, their perspectives while receiving medical services and, patients’ perceptions of the impact of the process of care may provide a different point of view regarding healthcare delivery [15].

The context of patient experience is multi-dimensional. Any feedback provided by patients regarding their perceptions of met needs after a clinical encounter or ward rounds is considered a component of the patient experience [16, 17]. Through applying patient-reported experience measures (PREMs), researchers or clinicians would be able to identify what patients value the most during patient-healthcare provider interaction and acknowledge feedback directly from patients regarding how to fine-tune provision of integrated care and improving outcomes [18]. Doyle et al. [10] outlined a framework from a cluster of terms related to the patient experience into *relational* and *functional* aspects. Relational aspects refer to the interactions between patient and healthcare provider. Empathy, respect, and building mutual trust are factors that enable providers to offer self-care interventions to patients and adequately engage them in their own decision-making. Functional aspects emphasize the logistics of healthcare delivery that entail the efficiency and effectiveness of healthcare services, smooth transition between facilities, clean and safe environment as well as physical access to healthcare services.

There is an increasing focus on capturing, measuring and analyzing the patient experience for a variety of high-volume conditions (including osteoarthritis, osteoporosis, and low back pain, and rheumatoid arthritis) [10, 19–26], as a means to drive better patient-centered care and improve the quality of healthcare delivery. There could be value in providing an overview of the patient experience when seeking care from healthcare providers and services in the healthcare system. An overview of reviews aims to appraise and summarize the evidence from multiple reviews on the same topic, which can support healthcare provider’s decision-making and facilitate the development of clinical guidelines [27].

Thus, the objectives of this overview of reviews is to 1) identify aspects of the patient experience when seeking care for musculoskeletal disorders from healthcare providers and the healthcare system. 2) identify which mechanisms are used to measure aspects of the patient experience. This overview focused on adults as it was considered challenging to collect patient-reported experience outcomes from pediatric populations. It was our interest to critically appraise, summarize, and identify gaps in the current evidence about the experiences of patients when seeking care from healthcare providers and services in the healthcare system.

## Methods

### Protocol and registration

The protocol of this overview of reviews is registered on the International Prospective Register of Systematic Reviews (PROSPERO: CRD42019136500). This overview of reviews was conducted and reported according to the Preferred Reporting Items for Systematic Reviews and Meta-Analyses (PRISMA) [28] checklist and the Cochrane Handbook of Systematic Reviews of Interventions (overview of reviews section) [29].

### Search methods for identification of reviews

A systematic literature search was conducted in electronic bibliographic databases: CINAHL, PubMed, EMBASE, and Scopus from their inception up to December 2019, without language restrictions. The search strategies were developed by the biomedical librarian (LL). Controlled vocabulary and keywords related to musculoskeletal disorders, patient experience, and reviews were combined for the search and were adjusted for each of the databases previously mentioned. The searches were re-run just before the final analyses and further studies retrieved for inclusion. In addition to the electronic database search, the authors conducted citation tracking on the reference list of included reviews to identify any potentially eligible reviews. Reviews meeting the inclusion criteria that were not originally included during the electronic search and citation tracking were manually selected. The full search strategy is outlined in Appendix 1. All citations were imported into Covidence Software and dual-screened by the authors.

### Criteria for considering reviews for inclusion

#### Population of interest

The population of interest were adults (≥18 years of age), with at least one type of musculoskeletal disorders (i.e., low back pain, neck pain, osteoarthritis, rheumatoid arthritis, fibromyalgia, surgical pain after joint replacement or spinal fusion, and osteoporosis). Reviews investigating participants with systemic or non-musculoskeletal pathology (e.g. tumors or infection) or pregnancy were excluded, since there would be different expectations for healthcare providers and services from these populations and other confounding factors such as life expectancy.

#### Study design and selection

Considering the substantial amount of existing evidence on the topic of interest, we decided to include systematic reviews (with or without meta-analysis) or scoping reviews that examined any related concepts that fall within the definition of “patient experience”. If an eligible study was published in a language outside the primary or secondary languages of the authorship team (English, Portuguese, Chinese and Spanish) all possible efforts would be made to get a translation; if that was not feasible the study was excluded. Articles that investigated healthcare delivery aspects were also included.

#### Outcomes of interest

For this review, we considered the patient experience as “the sum of all interactions that patients have with the healthcare system, including their care from health plans, and from doctors, nurses, and staff in hospitals, physician practices, and other healthcare facilities, shaped by an organization’s culture, that influence patient perceptions across the continuum of care” [30].

Doyle et al. [10] proposed compiling the patient experience into relational (*interpersonal*) and functional (*logistics of healthcare delivery*) aspects. We adopted this general framework into Table 1 in an attempt to identify patient experience for our target population.

**Table 1:** Modified themes of patients’ perceptions with musculoskeletal disorders regarding their experience with healthcare providers and health services

Relational aspects	Functional aspects
(1) Psychological and emotional support from healthcare providers with empathy, compassion, respect, and kindness	(1) Effective, timely and individualized treatment
(2) Healthcare providers understanding of patient expectations, values, beliefs, preferences, and concerns regarding their condition and treatment	(2) Patients’ perceptions of healthcare providers’ expertise, professional competence, and clinical skills
(3) Patients information needs of their conditions, treatment options, benefits, and harms	(3) Physical support and environmental needs (e.g., clean, safe, comfortable facilities)
(4) Involvement and engagement of patients and their family during decision-making process	(4) Continuity and coordination between transitions of care
(5) Transparent and clear communication between patients and healthcare providers focused on tone and honesty	(5) Privacy when seeking health services

#### Mechanisms used to measure relational and functional aspects of patient experience

We included several methods such as paper and electronic survey, focus group, patient journal, and interview, and patient-reported experience measures that have been utilized as instruments to measure and track changes of different aspects of patients’ perceptions.

### Selection of reviews

Four reviewers (working in groups of two: AC and MC, KB and BG) independently screened titles and abstracts to identify relevant studies for full texts based on the agreed eligibility criteria checklist and approved by the senior advisors (AG and CC). The same reviewers independently screened full texts for final inclusion. Any disagreement between reviewers was resolved by discussion and reaching consensus. If the initial reviewers failed to reach a consensus, a third reviewer from the other group arbitrated. Agreement between reviewers (on the independent inclusion of title/abstracts and full-text articles) were quantified using a kappa statistic [31, 32].

### Data extraction and management

Four reviewers (AC, MC, KB, and BG) independently extracted data from the included studies, using a standardized data extraction form. The following data were extracted: a) authors, year of publication, b) study design (systematic or scoping reviews), c) review country, d) settings of the individual studies, e) number and study designs of the individual studies, f) musculoskeletal disorder, g) relational and functional aspects of patient experience, h) data collection method, i) and main findings. Disagreement in the data extracted between reviewers was resolved by discussion and if necessary, arbitration by a third reviewer (AG).

### Assessment of methodological quality of included reviews

Four reviewers (AC, MC, KB, and BG) independently assessed the methodological quality of included studies using A MeaSurement Tool to Assess systematic Reviews (AMSTAR-2) [33]. AMSTAR-2 is a validated instrument that uses 16 questions to assess the quality of systematic reviews that include randomized and/or non-randomized studies of healthcare interventions. Reviewers rated either “yes” or “no” for each question based on the extent an article met certain criteria; and “partial yes” or “not applicable” for a few questions. The reviews were rated in overall confidence into four categories: “high”, “moderate”, “low”, and “critically low”, which was calculated using the AMSTAR checklist [34].

We considered critical domains of reviews, which included 1) whether or not protocol registered before commencement of the review, 2) the adequacy of the literature search, 3) the justification for excluding individual studies, 4) the methodological quality from individual studies being included in the review, 5) consideration of methodological quality when interpreting the results of the review and 6) the assessment of presence and likely impact of publication bias. It is not mandatory for scoping reviews to have a protocol, an article appraisal risk of bias tool, or syntheses of findings from individual studies, hence, when appraising scoping reviews with AMSTAR-2, all criteria related to any of these were considered not applicable [35]. Disagreements between the reviewers were resolved by discussion with the involvement of a third reviewer (AG) when necessary.

### Data synthesis

We used the PRISMA flow diagram to summarize the selection of reviews and summarized the characteristics of the included reviews in structured tables. Because the outcome data included in this review are not quantitative, the results of patient experience aspects were reported descriptively. We calculated the proportion of relational and functional aspects reported by the included reviews. Themes were identified and categorized based on the definition of aspects of patient experience outlined by Doyle et al. [10] (Table 1).

## Results

### Search results

**Fig. 1:**
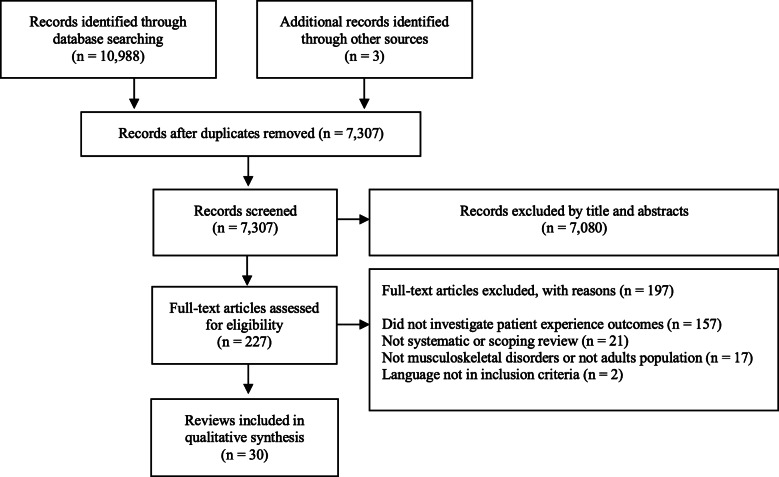
Study flow diagram

From the electronic search, 7307 potentially relevant articles were identified from four databases after the removal of duplicates based on titles and abstracts. Of these, 7080 were not relevant and 227 were retrieved in full texts. For the screening of titles and abstracts, the inter-rater agreement rate between the reviewers [(AC and MC) and (KB and BG)] resulted in a Cohen’s Kappa rate of 0.32 (fair agreement) and 0.51 (moderate agreement). The full-text review resulted in 30 included reviews [10, 19–26, 36–56] (Fig. 1). Of these, two [10, 36] were manually included when searching for relevant studies on PubMed and met the eligibility criteria; and one [54] was included after a manual search from reference lists of the included studies. The most common reasons for exclusion at the full-text reading stage were outcomes not related to our study purpose (*n* = 157), study designs that were neither systematic nor scoping reviews (*n* = 21), and other conditions not related to musculoskeletal disorders (*n* = 17) (Fig. 1). We have provided a list of relevant studies read in full-text, but excluded from the review with their respective reasons for exclusion (Appendix 2).

### Characteristics of the included reviews

All the included reviews were written in English and published between 2004 and 2019. The majority of the studies were conducted in Australia [19–22, 25, 26, 36, 41, 42, 52, 54], followed by the United Kingdom [10, 23, 24, 37, 40, 44–47, 49, 56] Canada [12, 48, 53, 55], the Netherlands [24, 38, 50], Ireland [41], Italy [53], Denmark [39], Belgium [24], and the United States [43]. There were twelve scoping reviews [19–22, 25, 26, 38, 46, 47, 52, 54, 55], eighteen systematic reviews [10, 23, 24, 36, 37, 39–45, 48–51, 53, 56] and six systematic reviews with meta-analyses [39–41, 49, 51, 53]. The numbers of the included studies for individual reviews ranged from 10 [37, 39, 40] to 323 [54]. Of all the available data, the combined numbers of participants in a single review ranged from 223 [37] to 31,791 [51]. The designs of the included studies varied among reviews, including qualitative, quantitative studies, mixed methods (qualitative and quantitative studies), cohort studies, cross-sectional and cohort studies (Table 2).

**Table 2:** Descriptive characteristics of the included reviews. (*n* = 30)

Review	Systematic or scoping review	Country	Settings	No. of studies	Designs of the included studies	Musculoskeletal disorder	Outcomes
Verbeek, 2004 [50]	Systematic review	The Netherlands	Not reported	20	12 qualitative, 8 quantitative	Non-specific low back pain	Relational and functional aspects
O’Neill, 2007 [40]	Systematic review and meta-synthesis	United Kingdom	Hospitals, a church or a senior center, or orthopedic surgeons waiting lists	10	All 10 articles are qualitative studies. Four of the studies applied a Grounded Theory approach to analyze the data; four adopted a Content Analysis approach, one applied Interpretative Phenomenology and one Interpretative Phenomenological Analysis.	Patients with osteoarthritis who either have already received total knee replacement or are on the waiting lists of knee replacement surgery or do not want to have surgery.	Relational and functional aspects
Slade, 2010 [23]	Systematic review	United Kingdom	Not reported	11	Not reported	Low back pain	Relational and functional aspects
Campbell, 2011 [44]	Systematic review	United Kingdom	Not reported	17	7 cohort studies, 10 cross-sectional studies	Non-specific spinal pain	Relational and functional aspects
Hush, 2011 [51]	Systematic review and meta-analysis	Australia	Private clinics, hospital outpatient clinics, spine clinics, and an athlete rehabilitation clinic	15	9 cross-sectional patient surveys, 2 clinical trials, 1 longitudinal cohort study, and 3 qualitative studies	Seven studies investigated patients with mixed musculoskeletal or soft tissue injuries, 6 studies investigated patients with back pain, and one study investigated athletes with lower-limb injuries.	Relational and functional aspects
Doyle, 2013 [10]	Systematic review	United Kingdom	Primary and secondary care including hospitals and primary care centers.	55	15 systematic reviews/meta-analysis, 40 individual studies	Varied (cardiac, cancer, diabetes, pulmonary, acute, hypertension, chronic, pain, mental health, general, other)	Relational and functional aspects
Hopayian, 2014 [47]	Scoping review	United Kingdom	Spinal triage service, general practice, pain clinic, back clinic (osteopath and acupuncturist), physiotherapy, X-ray department, physiotherapy, and acute care services, chiropractic, university campus, community, back pain rehabilitation	28	Qualitative studies, mixed-method studies, questionnaire surveys using open questions to collect and interpret data qualitatively, and qualitative studies that were parallel to or imbedded in trials or observational studies.	Low back pain, sciatica	Relational and functional aspects
Slade, 2014 [49]	Systematic review and meta-analysis	United Kingdom	Not reported	15	15 qualitative studies	Chronic non-specific chronic low back pain	Relational and functional aspects
Zuidema, 2015 [38]	Scoping review	The Netherlands and Belgium	Not reported	17	Cross-sectional studies, and a single group longitudinal design	Rheumatoid arthritis	Relational and functional aspects
Fu, 2016 [37]	Systematic review	United Kingdom	Not reported	10	Not reported	Chronic back pain	Relational and functional aspects
O’Keeffe, 2016 [41]	Systematic review and meta-synthesis	Ireland and Australia	Not reported	13	5 used semi-structured interview, 5 used focus group, 1 used Cross-case analysis/interview, 1 used nominal group technique interview, and 1 used mixed-methods	Patients with subacute, chronic, non-specific or intermittent low back pain, neck pain, or musculoskeletal conditions. Physical therapists working in primary care, for patients who have undergone torture or specializing in Norwegian psychomotor physical therapy	Relational and functional aspects
McMurray, 2016 [48]	Systematic review	Canada	Outpatient rehabilitative care, inpatient rehabilitative care hospital, rehabilitation in acute care hospitals and hospital to the community	33	14 used a quantitative method, 2 used survey, 10 used Cross-sectional, 4 used mixed methods, 1 used comparative psychometric testing, 1 used randomized controlled trial and 1 is a descriptive, structured literature review.	Heterogeneous, described as those characterized by issues with musculoskeletal disorders, stroke/neurology, frail/older adults and medical complexity, multiple sclerosis, occupation-related musculoskeletal disorders, cardiopulmonary disorders, or rheumatologic disorders or were discharged patients, inpatients, patients with stroke and their caregivers, patients and their physicians, patients, patients receiving unspecified rehabilitative care, or amputees	Relational and functional aspects
Wluka, 2016 [54]	Scoping review	Australia	Not reported	323	Not reported	Inflammatory arthritis specifically rheumatoid arthritis and ankylosing spondylitis, osteoarthritis, back pain, neck pain, and osteoporosis	Relational and functional aspects
Chou, 2017 [21]	Scoping review	Australia	Rheumatology clinics, outpatient screening unit at a teaching hospital, fracture clinic of a large teaching hospital, health maintenance organization, centers performing bone densitometry, outpatient clinics in osteoporosis centers, emails, advertisements in a tertiary hospital medical center newsletter, National Osteoporosis Society support groups, osteoporosis exercise classes, South Asian community centers, urban fracture clinic, academic primary care sites	33	19 studies used quantitative methods, 14 used qualitative methods	Osteoporosis (patients were classified as having osteoporosis based on bone densitometry in 7 studies, requiring prescription medications in 6 studies or based on previous fragility fractures or high risk of osteoporotic fractures in 8 studies. The diagnosis of osteoporosis or osteopenia was unspecified in 13 studies)	Relational and functional aspects
Papandony, 2017 [22]	Scoping review	Australia	Public and private hospitals, acupuncture clinics, pharmacies, outpatient orthopedic clinic at local hospital, private general practice clinic, retirement, own home, primary care community clinics, solo practitioners’ walk-in clinics, hospital-based family medicine units, participants from long-term studies, ambulatory care clinic, regional orthocenter, single surgeon practice, university medical center	21	9 studies used quantitative methods, including written questionnaires, computer questionnaires or interviews. 12 studies used qualitative methods including focus groups and individual interviews. 1 study employed both quantitative and qualitative methods with interviews, patient diaries, and group teaching sessions	Osteoarthritis	Relational and functional aspects
Wijma, 2017 [24]	Systematic review	Belgium and the Netherlands	Private physiotherapy practices, health sciences center in a university, respondent’s or researcher’s workplace, home, onsite observation in an academic medical center, or national health service hospital; physiotherapy practices, rehab centers in various countries	14	4 used grounded theory; 1 used nominal group technique; 2 used ethnography; 1 used a descriptive qualitative approach; 1 used phenomenography; 2 used phenomenology; and 3 have no specific design	Studies recruited participants not limited to patients with musculoskeletal disorders as well as therapists working in various fried of rehabilitation setting	Relational and functional aspects
Hulen, 2017 [43]	Systematic review	United States	Hospitals, rehab centers, clinics	22	Qualitative (*N* = 12), quantitative (*N* = 9) and mixed-methods (*N* = 1) designs	Rheumatoid arthritis	Relational and functional aspects
Gillespie, 2017 [46]	Scoping review	United Kingdom and Canada	Pre-hospital care, acute medical ward, Medical Specialties, Obstetric Care, Hospital Care, geriatric care, General practice, Palliative care, Outpatient physiotherapy/ rehabilitation services, Outpatient physiotherapy/ rehabilitation services, Careers/Cancer service uses/ older people/ men’s health/ parents/ human immunodeficiency virus service users, Fertility clinic, Intensive care unit, Community hospice programs, Oncology, Primary care, Ambulatory care, Academic medical center, Psychiatric care, Lymphoma care, Geriatric ward, Palliative care, illicit drug users, regional hospital	44	Primary and secondary studies using qualitative, quantitative, and mixed-methods designs	Not reported	Relational and functional aspects
Chou, 2018 [19]	Scoping review	Australia	Family care center in Memorial Hospital, general practitioner practices, outpatient clinics, chiropractic offices, physical therapy offices and departments, advertisements, community hospitals, rehab centers, pain centers, campus-wide emails and word of mouth, poster advertisements, senior centers, spinal clinics, and computerized databases	43	30 qualitative, 12 quantitative, and 1 mixed-methods	Non-specific low back pain, with or without leg pain, excluding back pain from fractures, malignancy, infection, and inflammatory spinal disorders.	Relational and functional aspects
Chou, 2018 [20]	Scoping review	Australia	Hospitals, rehab centers, clinics	50	35 qualitative, 14 quantitative, 1 mixed-methods study	Chronic low back pain	Relational aspects
Chou, 2018 [25]	Scoping review	Australia	Not reported	30	16 qualitative, 11 quantitative and 3 mixed-methods studies	Osteoarthritis using American College of Rheumatology criteria in 3 studies, radiographic change and pain in 4 studies, self-report in 6 studies, chart review in 3 studies, clinical diagnosis in 4 studies, and by undefined methods in 8 studies	Relational aspects
Segan, 2018 [26]	Scoping review	Australia	Hospital outpatient rheumatology clinics, nurse-led university hospital clinics, medical centers, the United States of America National Psoriasis Foundation, private rheumatology clinics, patients obtained through internet and email, patients recruited from outpatient rheumatoid arthritis clinics from National Health Service trusts, patients recruited from members of the United Kingdom National Rheumatoid Arthritis Society, private practices, patients recruited from arthritis database,	27	16 qualitative, 9 quantitative, 2 mixed-methods	Inflammatory arthritis	Relational and functional aspects
Rothmann, 2018 [39]	Systematic review and meta-synthesis	Denmark and Australia	Not reported	10	Individual interviews consisting of both face-to-face and telephone interviews (*n* = 9) and focus groups (*n* = 1).	Osteoporosis & Individuals with at least one risk factor of osteoporosis & t-score ≤ − 2.5 or a fragility fracture & Individuals aged 45 years and above	Relational aspects
Raybould, 2018 [45]	Systematic review	United Kingdom	Secondary care populations, primary care, community, or mixed settings.	16	11 single semi-structured interviews and 6 focus groups	Osteoporosis, vertebral fracture, osteopenia.	Relational and functional aspects
Chou, 2018 [52]	Scoping review	Australia	Not reported	44	25 qualitative, 18 quantitative and 1 mixed-methods study	Low back pain	Relational and functional aspects
Rossettini, 2018 [53]	Systematic review and meta-synthesis	Italy and Canada	Rheumatology outpatient clinics, inpatient wards, disease registries or databases, hospital outpatient clinics, private or community rheumatology clinics, inpatient, outpatient, databases	11	Not reported	Individuals experiencing musculoskeletal pain defined as the consequence of everyday activities that repeatedly or unusually stress the system, or due to either acute traumatic events or to chronic complaints	Relational and functional aspects
Lim, 2019 [36]	Systematic review	Australia	Primary care practice, tertiary pain clinics, hospital or rehabilitation clinics, specialist spine or osteopathy clinics, general community, research centers, education forum, occupational health clinic	41	33 qualitative, 5 quantitative and 3 used mixed methods	Non-specific low back pain, with or without leg pain, excluding back pain related to fractures, malignancy, infection, and inflammatory back conditions	Relational and functional aspects
Connelly, 2019 [42]	Systematic review	Australia	Hospitals, rehab centers, clinics	29	11 quantitative, 14 qualitative and 4 mixed methods	Rheumatoid arthritis, ankylosing spondylitis, psoriatic arthritis, reactive arthritis, and other types of unspecified inflammatory arthritis	Relational and functional aspects
Asif, 2019 [55]	Scoping review	Canada	Transitions of patients between settings (e.g., transfer of patient from an acute care facility to a nursing home, or rehabilitation to home)	11	Most included studies used a qualitative design (*n* = 10), with only one quantitative study	Hip fracture	Functional aspects
Davenport, 2019 [56]	Systematic review	United Kingdom	Outpatient/community and an inpatient stay.	18	10 qualitative design, 1 convergent mixed methods design, 1 interpretive phenomenology, 1 focused ethnographic design, 5 did not state a theoretical approach	Mixed, stroke (*n* = 3), head and neck cancer (*n* = 2), mixed rehabilitation (*n* = 2), various speech pathologies (*n* = 1), low back or neck pain (*n* = 7), jaw pain (*n* = 1), chronic fatigue/ myalgic encephalomyelitis (*n* = 1) and older adults post hip fracture (*n* = 1)	Relational aspects

Each review targeted various health conditions and populations such as non-specific low back pain [19, 20, 23, 36, 37, 41, 44, 47, 49, 50, 52] (*n* = 11 reviews, *n* = 37,408 participants), osteoporosis [21, 39, 45] (*n* = 3 reviews, *n* = 17,534 participants), osteoarthritis [22, 25, 40] (*n* = 3 reviews, *n* = 3157 participants), rheumatoid arthritis [26, 38, 42, 43] (*n* = 4 reviews, *n* = 9406 participants), and other musculoskeletal disorders (e.g., chronic pain, soft tissue injuries, lower-limb sports-related injuries, traumatic musculoskeletal injuries, mixed and unspecified) [10, 24, 46, 48, 51, 53–56] (*n* = 9 reviews, *n* = 61,772 participants) that sought unspecified physical therapy services or rehabilitative cares. Table 2 provides an overview of the characteristics of the included reviews.

### Methodological quality of included reviews

As reported in Table 3, the majority of the included reviews (*n* = 16 reviews) [19–22, 24–26, 38, 39, 42, 46, 47, 52–55] have “moderate” quality, which were determined based on AMSTAR-2; with the remainders rated as “critically low” (*n* = 6 reviews) [10, 23, 37, 40, 48, 56] or “low” quality (*n* = 8 reviews) [36, 41, 43–45, 49–51]. In this study, questions 2, 9, and 13 in AMSTAR-2 were considered not applicable for scoping reviews because a written protocol and a risk of bias assessment are not mandatory in scoping review designs [35]. All of the reviews specified their population of interest and main outcome (question 1) and all listed their databases, keywords, and inclusion/exclusion criterion in their search strategies (questions 2 and 4). Eighteen (60%) out of the 28 reviews [19–22, 25, 36, 38, 41, 43, 44, 46–49, 52, 53, 55, 56] involved at least two reviewers independently performed study selection (question 5) and eighteen (60%) of them [20–22, 25, 36, 38, 41–43, 45–51, 53, 56] involved at least two reviewers independently performing data extraction and reaching consensus (question 6). For question 8, most of the reviews (*n* = 27, 90%) [10, 19–22, 24–26, 36, 39–56] described and organized their included studies in adequate detail, providing information such as population, outcomes, research designs, and study settings. For systematic reviews, 12 of them [24, 36, 37, 39, 41, 42, 44, 45, 49, 53, 56] utilized the risk of bias assessment tools to appraise included studies. Questions 11, 12, and 15 are designed specifically for meta-analysis. All of the reviews with meta-analyses (*n* = 6, 20%) [39–41, 49, 51, 53] included in this study used appropriate methods for statistical combination of results, but none of them reported the potential impact of risk of bias in individual studies on the results nor carried out an adequate investigation of publication bias. None of the reviews reported the source of funding for the individual studies (question 10).

**Table 3:** Methodological quality of included reviews. (AMSTAR-2^a^)

Reviews	Q1	Q2^c^	Q3	Q4	Q5	Q6	Q7	Q8	Q9^c^	Q10	Q11^c^	Q12^c^	Q13^c^	Q14	Q15^c^	Q16	Quality^b^
**Scoping reviews**																	
Hopayian, 2014 [47]	Y	NA	N	PY	Y	Y	N	PY	NA	N	NA	NA	NA	Y	NA	Y	Moderate
Zuidema, 2015 [38]	Y	NA	N	PY	Y	Y	N	N	NA	N	NA	NA	NA	Y	NA	Y	Moderate
Wluka, 2016 [54]	Y	NA	N	PY	N	N	N	PY	NA	N	NA	NA	NA	Y	NA	Y	Moderate
Chou, 2017 [21]	Y	NA	N	PY	Y	Y	N	Y	NA	N	NA	NA	NA	Y	NA	Y	Moderate
Papandony, 2017 [22]	Y	NA	N	PY	Y	Y	N	Y	NA	N	NA	NA	NA	Y	NA	Y	Moderate
Gillespie, 2017 [46]	Y	NA	N	PY	Y	Y	N	PY	NA	N	NA	NA	NA	Y	NA	Y	Moderate
Chou, 2018 [19]	Y	NA	N	PY	Y	N	N	Y	NA	N	NA	NA	NA	Y	NA	Y	Moderate
Chou, 2018 [20]	Y	NA	Y	PY	Y	Y	N	Y	NA	N	NA	NA	NA	N	NA	Y	Moderate
Chou, 2018 [25]	Y	NA	Y	PY	Y	Y	N	Y	NA	N	NA	NA	NA	Y	NA	Y	Moderate
Segan, 2018 [26]	Y	NA	Y	PY	N	N	N	PY	NA	N	NA	NA	NA	Y	NA	Y	Moderate
Chou, 2018 [52]	Y	NA	Y	PY	Y	N	N	PY	NA	N	NA	NA	NA	Y	NA	Y	Moderate
Asif, 2019 [55]	Y	NA	N	PY	Y	N	N	PY	NA	Y	NA	NA	NA	Y	NA	Y	Moderate
**Systematic reviews**																	
Verbeek, 2004 [50]	Y	N	N	PY	N	Y	N	PY	N	N	NA	NA	N	Y	NA	Y	Low
Slade, 2010 [23]	Y	N	Y	PY	N	N	N	N	N	N	NA	NA	N	Y	NA	Y	Critically low
Campbell, 2011 [44]	Y	N	Y	PY	Y	N	N	PY	Y	N	NA	NA	N	Y	NA	Y	Low
Doyle, 2013 [10]	Y	PY	N	N	N	N	N	Y	N	N	NA	NA	N	Y	NA	Y	Critically low
Fu, 2016 [37]	Y	N	N	PY	N	N	N	N	N	N	NA	NA	Y	Y	NA	Y	Critically low
Hulen, 2016 [43]	Y	N	Y	PY	Y	Y	PY	PY	N	N	NA	NA	N	N	NA	Y	Low
McMurray, 2016 [48]	Y	N	N	Y	Y	Y	N	Y	N	N	NA	NA	N	Y	NA	Y	Critically low
Wijma, 2017 [24]	Y	PY	Y	Y	N	N	N	Y	Y	N	NA	NA	Y	Y	NA	Y	Moderate
Raybould, 2018 [45]	Y	N	N	PY	N	Y	N	PY	PY	N	NA	NA	N	N	NA	Y	Low
Lim, 2019 [36]	Y	PY	Y	PY	Y	Y	N	PY	Y	N	NA	NA	Y	Y	NA	Y	Low
Connelly, 2019 [42]	Y	PY	Y	PY	N	Y	Y	PY	Y	N	NA	NA	Y	N	NA	Y	Moderate
Davenport, 2019 [56]	Y	N	Y	PY	Y	Y	N	PY	N	Y	NA	NA	N	Y	NA	Y	Critically low
**Systematic reviews with meta-analysis**																	
O’Neill, 2007 [40]	Y	N	N	N	N	N	N	Y	N	N	Y	N	N	Y	N	Y	Critically low
Hush, 2011 [51]	Y	PY	Y	PY	N	Y	N	PY	N	N	Y	N	Y	Y	N	N	Low
Slade, 2014 [49]	Y	N	Y	PY	Y	Y	N	PY	Y	N	Y	N	N	N	N	Y	Low
O’Keeffe, 2016 [41]	Y	Y	Y	Y	Y	Y	N	PY	PY	N	Y	N	N	Y	N	Y	Low
Rothmann, 2018 [39]	Y	Y	N	PY	N	N	N	PY	PY	N	Y	N	Y	Y	N	Y	Moderate
Rossettini, 2018 [53]	Y	Y	Y	PY	Y	Y	Y	Y	Y	N	Y	Y	Y	Y	N	Y	Moderate

^a^A MeaSurement Tool to Assess systematic Reviews (AMSTAR-2) tool. ^b^calculated by AMSTAR-2 checklist [36] *Y* yes, *N* no, *PY* partial yes, *NA* not applicable. ^c^Q2, Q9, Q13 are not applicable for scoping reviews, and Q11, Q12, and Q15 are only applicable for studies with meta-analysis

Q1. Did the research questions and inclusion criteria for the review include the components of PICO? Q2. Did the report of the review contain an explicit statement that the review methods were established prior to the conduct of the review and did the report justify any significant deviations from the protocol? Q3. Did the review authors explain their selection of the study designs for inclusion in the review? Q4. Did the review authors use a comprehensive literature search strategy? Q5. Did the review authors perform study selection in duplicate? Q6. Did the review authors perform data extraction in duplicate? Q7. Did the review authors provide a list of excluded studies and justify the exclusions? Q8. Did the review authors describe the included studies in adequate detail? Q9. Did the review authors use a satisfactory technique for assessing the risk of bias in individual studies that were included in the review? Q10. Did the review authors report on the sources of funding for the studies included in the review? Q11. If meta-analysis was performed did the review authors use appropriate methods for statistical combination of results? RCTs Q12. If meta-analysis was performed, did the review authors assess the potential impact of RoB in individual studies on the results of the meta-analysis or other evidence synthesis? Q13. Did the review authors account for RoB in individual studies when interpreting/ discussing the results of the review? Q14. Did the review authors provide a satisfactory explanation for, and discussion of, any heterogeneity observed in the results of the review? Q15. If they performed quantitative synthesis did the review authors carry out an adequate investigation of publication bias (small study bias) and discuss its likely impact on the results of the review? Q16. Did the review authors report any potential sources of conflict of interest, including any funding they received for conducting the review?

### Patient experience with healthcare providers and health services outcomes (Table 4)

There was a broad range of patient experience aspects reported by the included reviews. As stated in the methods, we considered patient experience from the perspective of relational and functional aspects. All reviews except one [55] reported patient experience outcomes from the perspective of relational aspects (*n* = 29, 97%), 26 reviews (87%) [10, 19, 21–24, 26, 36–38, 40–55] from the perspective of functional aspects; and 25 (83%) [10, 19, 21–24, 26, 36–38, 40–54] of the reviews included both relational and functional aspects. Among the included reviews, only three [41, 51, 53] specifically investigated the interactions between patients and physical therapists. The majority of the included reviews stood from the patient’s perspective focusing on a certain musculoskeletal diagnosis, and they reported the overall patient experience while seeking healthcare services regardless of the providers they encountered.

**Table 4:** Identified themes of the patient experience from included reviews

Patient Experience Outcomes	Measure (data collection method)	No. of reviews (%)	Musculoskeletal disorders	Findings
**Relational**				
Psychological support	Survey, questionnaires, interviews, telephone interviews, focus groups, narrative methods, mixed methods	13 (43%)	Non-specific low back pain [19, 23, 36, 44], Osteoporosis [39], Rheumatoid arthritis [26, 38], Others [10, 24, 46, 53, 54, 56]	Establishes rapport, enables emotional comfort, enables connectedness [10, 38, 44, 46, 53, 54], relieving fear and anxiety, treated with kindness, dignity, compassion and positive attitude [10, 46, 56], emotionally supportive, encouraging and patient-centered healthcare [19, 24, 36, 46], potential psychological and social consequences of the diagnosis [39], knowing you can get help when you need it is important [39], previous negative experiences with medical consultations [26], ethical practice [23]
Understanding (patient expectations)	Survey, questionnaire, interviews, telephone interviews, focus groups	18 (60%)	Non-specific low back pain [19, 23, 37, 41, 47, 49, 50, 52], Osteoporosis [21, 39], Osteoarthritis [40], Rheumatoid arthritis [26], Others [10, 24, 48, 51, 53, 56]	Respect, being listened to, empathy, mutual understanding [10, 19, 23, 26, 37, 39, 48–51, 56], getting to know the patient [24, 49], Taking patient opinion and preference into consideration [21, 23, 41], desirable characteristics of the medical practitioners (being non-judgmental, non-egotistical with an open interested attitude and mind, honest about his/her limitations and reflective of his/her own behavior and emotions, friendly, supportive, considerate, patient, genuine, polite, positive, caring for the patient, the ability to care for the patient, taking the patient seriously, believing in the patient, recognition of the patients’ emotions, making a commitment to the patient, and making the best effort, enables connectedness, punctual, reliable, transparent, open to second opinion, fully informing, and welcomes questions) [21, 23, 24, 47, 53], expectation of treatment (participants had negative perceptions of surgery because of the associated risks, previous positive or negative experiences with physiotherapy and their treatment of their clinical condition) [40, 53], expectation of condition (Patients have the perception that “there are probably people worse off” and they should have priority for surgery) [40], Congruent patients experienced more pain relief and effectiveness of the treatment than noncongruent patients [50]., Patients believed that physiotherapy-delivered care helped with pain relief, facilitated a better understanding of pain management strategies, prevented worsening of low back pain and improved mobility and function [52], Chiropractic therapy was perceived by some patients to be effective; however, others were concerned about adverse outcomes [52].
Information needs (education)	Cross-sectional surveys, questionnaire, interviews, telephone interviews, focus groups, diaries, video recording	24 (80%)	Non-specific low back pain [19, 20, 23, 36, 37, 41, 44, 47, 49, 50], Osteoporosis [21, 39, 45], Osteoarthritis [20, 22], Rheumatoid arthritis [26, 38, 42], Others [10, 24, 51, 53, 54, 56]	Patients’ perceived need to obtain health information from a variety of sources and health information content about the diseases [19, 20, 25, 36, 38, 44, 45, 54], perceived needs for imaging for diagnostic purposes and legitimation of symptoms [20, 36, 50], explaining the patient’s condition such as possible symptoms and cardiovascular risks and educating the patient about treatments, self-management strategies and physical exercises [10, 22–24, 36, 38, 39, 41, 42, 45, 47, 49–54, 56], reasons for seeking health information, delivery modes and barriers to meeting health information needs [36, 37, 42], information needs and concerns about medications [20–22, 38, 45], clear, comprehensive information that raises awareness of available options, risks, and benefits of treatments [10, 54], lack of information enhance worries [39], a minor health concern using the comparison to gain a sense of osteoporosis [39], patients with low back pain sought healthcare from medical practitioners to obtain a diagnosis, receive management options, sickness certification and legitimation for their low back pain. However, there was dissatisfaction with the cursory and superficial approach of care [20], patients’ perceived need of invasive therapies (patients avoided injections and surgeries) [20], Desired information content was broad, and included targeted and practical information covering disease treatment and psychosocial wellbeing [42], written and verbal information [45], necessity of diagnosis [47], patients’ need to gain information by sharing experiences with other patients [26], understanding the prognosis [54], specific information, tailored to their condition, rather than generalities [54]
Shared decision-making (patient involvement and engagement)	Survey, questionnaire, interviews, telephone interviews	12 (40%)	Non-specific low back pain [19, 23, 37, 50], Osteoarthritis [40], Rheumatoid arthritis [26], Others [10, 24, 48, 51, 54, 56]	Shared decision-making [19, 26, 50, 51, 53, 54], patient involvement [37], patient engagement (Fully informed, provided with test results, prognosis explained, given self-help strategies, preventative strategies, home program, responds to feedback, choice of provider, choice of treatment, communication with other care providers/health professionals, play an active role in their management) [23, 48, 54, 56], patient empowerment [24](8), Involvement of, and support for family and caregivers in decisions [10, 23, 48], Working with patient-defined goals [24, 48], symptoms and information sources were the two main factors influencing patient decision-making [40], partnership of care [53]
Communication	Survey, questionnaire, interviews, telephone interviews, dairies	16 (53%)	Non-specific low back pain [19, 36, 37, 41, 47, 49, 50], Osteoarthritis [22], Rheumatoid arthritis [26, 43], Others [10, 24, 51, 53, 54, 56]	Good communication skills [19, 22, 26, 37, 49–51, 53, 54, 56], language and tone used [36], transparency, honesty, disclosure when something goes wrong [10], continuous tailored communication in lay speech [24], non-verbal communication [24], interpersonal skills: listening, empathy, friendliness, encouragement, confidence [41, 43, 47]
**Functional**				
Effective, individualized treatment	Survey, questionnaire, interviews, telephone interviews, diaries	12 (40%)	Non-specific low back pain [37, 41, 47, 49, 50, 52], Osteoarthritis [22], Rheumatoid arthritis [43], Others [10, 24, 51, 53]	Individualized, patient-centered care [22, 24, 37, 41, 47, 49, 51, 52], Timely, tailored and expert management of physical symptoms [10, 51], achieving normalcy and wellness maintenance, complete recovery, pain control and desirable outcomes [43, 50, 53], expectations for pharmacological treatment that involved decreased side effect [43], perceived needs for choice of treatment options such as pharmacologic therapy and pain management methods, complementary and alternative medicine (CAM), joint replacement surgery, orthoses and physical aids [22] Pain relief can be regarded as the driving force for seeking treatment or for returning for subsequent treatment [50].
Trusted expertise	Cross-sectional surveys, questionnaire, interviews, telephone interviews, diaries, video recording, focus groups	16 (53%)	Non-specific low back pain [19, 23, 37, 41, 47, 50], Osteoporosis [21, 45], Osteoarthritis [22, 40], Rheumatoid arthritis [38], Others [10, 24, 46, 53, 54]	Perception of the health professionals’ role [40, 54], qualifications, competence, and technical skills [19, 22, 23, 46, 53], physical therapist practical skills, expertise, knowledge, and training [24, 41], perceived physician knowledge and attitudes and beliefs [45], patients’ perceived needs of investigations for diseases [21, 50], the need for thorough assessment and holistic care [19, 22, 50], the need for a diagnosis and finding a cause of pain [19, 50], trusted professionals [10, 47], role of the health professionals as being important in helping them find solutions to cope with their pain, holding them accountable for pain management [37], validation by the multidisciplinary panel [38], confidence [24, 50]
Physical and environmental needs (social support)	Survey, questionnaire, interviews, diaries	16 (53%)	Non-specific low back pain [23, 36, 41, 44], Osteoporosis [45], Osteoarthritis [22, 40], Rheumatoid arthritis [26, 38, 42, 43], Others [10, 48, 51, 53, 54]	Social connectedness, context and social support [36, 38, 43–45, 53], organizational factors, time, flexibility and simplicity with patient appointments and care [23, 41, 48, 53], attention to physical support needs and environmental needs (ex. clean, safe, comfortable, accessible environment) [10, 23, 26, 48, 51, 53, 54], convenient clinic hours, location, and parking, as well as available and approachable support staff [23, 51], practical support needs of adaptive workplace, living environment modification and coping strategies on how to continue daily activities and manage social roles by using assistive devices or aids [22, 23, 38, 54], the total knee replacement outcome was viewed positively or negatively when viewed concerning the participant’s life context or environment [40], group sessions had advantages for psychosocial issues while written information provided useful supplementation [42], financial and time cost [22, 51]
Continuity of care	Survey, questionnaire, interviews, diaries	13 (43%)	Non-specific low back pain [19, 37, 41, 44, 47, 50], Osteoarthritis [22], Rheumatoid arthritis [26, 42], Others [10, 46, 51, 55]	Feasibility and availability of healthcare service [37, 44], coordination and continuity of care; smooth transitions from one setting to another (patients and their caregivers may experience a lack of clarity about where clinical responsibilities ended and caregiver responsibilities began) [10, 19, 46, 47, 50, 55], the need for collaboration between different HCPs, confusion about the role of different healthcare providers [19, 55], time length of consultations and flexibility with patient appointments and care [26, 41, 50], barriers to meeting health information needs were around timely access [42], preferences for follow-up care [26, 51], timing and accessibility of appropriate care and in times of need [26], need for allied health and CAM [26], disorganized discharge planning (a focus on rapid discharge, absence of patient and caregiver involvement during discharge planning and a lack of standardized patient assessment during care transitions) [55], lack of information sharing with patients and caregivers included an absence of the following: healthcare providers-initiated conversations about treatment plans, accurate information about the recovery and information from hospital staff during discharge and admission [55].
Privacy	Survey, questionnaire	3 (10%)	Non-specific low back pain [23], Others [48, 51]	Respect for patient privacy [23, 48], lack of privacy will lead to less patient satisfaction [51]

### Relational aspects of patient experience outcome (Table 4)

In this overview of reviews, we found 13 (43%) reviews [10, 19, 23, 24, 26, 36, 38, 39, 44, 46, 53, 54, 56] which investigated psychological support for patient emotions and respectfully provide comfort and soothing fear and anxiety; 18 (60%) [10, 19, 21, 23, 24, 26, 37, 39–41, 47–53, 56] discussed healthcare providers’ understanding of patient expectations with respect of their beliefs and values; 24 (80%) [10, 19–24, 26, 36–39, 41, 42, 44, 45, 47, 49–51, 53, 54, 56] demonstrated the importance of patients’ perceived information needs that could be fulfilled by patient education; 12 (40%) [10, 19, 23, 24, 26, 37, 40, 48, 50, 51, 54, 56] entailed shared decision-making by involving and engaging patients and their families as part of crucial patient experience when receiving healthcare services; and 16 (53%) [10, 19, 22, 24, 26, 36, 37, 41, 43, 47, 49–51, 53, 54, 56] presented communication that minimizes the perceived information imbalance or gap between patients and healthcare providers, as relational aspects, that entail interpersonal skills during healthcare providers’ delivery of care.

### Functional aspects of patient experience outcome (Table 4)

According to our findings, 12 (40%) [10, 22, 24, 37, 41, 43, 47, 49–53] of the included reviews took effectively, individualized treatment delivered in a timely manner into consideration as functional aspects of patient experience; 16 (53%) [10, 19, 21–24, 37, 38, 40, 41, 45–47, 50, 53, 54] talked about trusted expertise and perceived social roles, traits, and characteristics of healthcare providers; 16 (53%) [10, 22, 23, 26, 36, 38, 40–45, 48, 51, 53, 54] discussed physical and environmental needs including access to healthcare and social support; 13 (43%) [10, 19, 22, 26, 37, 41, 42, 44, 46, 47, 50, 51, 55] introduced continuity of care, coordination in interdisciplinary healthcare team and smoothness of transition; and only 3 (10%) [23, 48, 51] mentioned privacy.

### Mechanisms used to measure patient experience aspects (Table 4)

Individual interviews were the most commonly used (*n* = 23 reviews) mechanism to collect data [10, 19–22, 24–26, 36, 37, 39–43, 45–47, 50–53, 56], followed by focus groups [19–21, 24–26, 36, 37, 39–43, 45–48, 50, 52, 53, 56], survey [10, 19–21, 25, 26, 42, 43, 47, 48, 51, 53], PREMs questionnaires [19–21, 25, 26, 36, 42, 43, 47, 48, 50, 56], phone interviews [20, 26, 39, 42, 45, 46, 50, 56] and diaries [25].

## Discussion

The main purpose of this study was to investigate the experience of people with musculoskeletal disorders when seeking healthcare services and their perception of healthcare providers. While considering abstract concepts about the patient experience, delineation and definition of relational and functional aspects provide a useful framework to scrutinize different themes and constructs in this field of study. In this overview of reviews, we identified five key themes in both the relational and functional aspects. In relational aspects, patients’ needs for education on and explanations about their conditions and interventions were of most prevalent findings [10, 22–24, 36, 38, 39, 41, 42, 45, 47, 49–54, 56]. In functional aspects, patients reported receiving effective individualized treatment [22, 24, 37, 41, 47, 49, 51, 52], and attention to physical support, such as expecting a clean, safe, comfortable, accessible clinical environment was important [10, 23, 26, 48, 51, 53, 54]. Based on our findings, we feel that there are key messages that need to be discussed.

### Relational aspects of patient experience

The patients’ understanding of their health condition and appropriate management highly depends on their health literacy [57]. Education about the natural course of certain diagnoses, multiple domains that drive pain and disability, as well as the psychosocial aspect of the pain experience, is recommended during patient-healthcare provider encounters [58]. It is also reported that delivering clear information with good communication skills would help patients cope with their health conditions and prognoses, which would facilitate establishing a trustworthy patient-healthcare provider relationship [47, 59].

Effective communication helps healthcare providers develop a clearer idea of patients’ feelings and their needs [53]. Meanwhile, patients would have an increased understanding of the scope and impact of their musculoskeletal disorder(s) and possible treatment options [54]. Such processes facilitate shared decision-making models whereby patients are empowered to participate in their medical management [26, 60]. Furthermore, psychological support can be influential, especially for those suffering from chronic pain, in diminishing possible fear-avoidance of initiating movement as well as compliance with their exercises throughout healthcare-seeking [61]. Therefore, to better promote quality and outcomes in healthcare, providers should consider improving their interpersonal skills to address the relational aspects of patient experience.

### Functional aspects of patient experience

First, the application of individualized, tailored treatment has been proposed in the management of musculoskeletal disorders, with emphasis on customizing interventions for any given individual pathological, functional, and psychosocial variations [62–64]. In a shared decision-making model, patient’s diagnoses, clinical manifestations, severity of symptoms, cognitive and mental status as well as their needs should all be taken into consideration to formulate holistic, personalized plans of care [65]. Second, continuity during transitions among different healthcare settings, and physical access to healthcare should also be addressed in integrated care [66]. Providing downstream transfer services after discharging patients from acute or subacute hospitals to rehabilitation facilities, nursing homes, or outpatient clinics is recommended to ensure that patients receive required medical attention and care without disruptions.

Third, physical accessibility of healthcare sites influences the patient experience. Environmental factors including commute distance [51], cleanliness, and barrier-free designs in clinics need to be considered. The flexibility of scheduling [23] and the complexity of paperwork also impact patients’ overall impression of healthcare facilities. Fourth, patients perceive the professional role [40, 54] of healthcare providers based on their qualifications, competence, technical skills [19, 22, 23, 46, 53], attitudes, and beliefs [45]. Patient’s trust in expertise is built upon the foundation of the knowledge and training [24, 41] of a healthcare provider as well as the validation by multidisciplinary healthcare team [38]. Finally, patients expect that their privacy should be fully respected before, during and after receiving health services [23, 48].

### Mechanisms of collecting patient experience

While efforts have been made to collect and measure information about the patient experience using qualitative studies or surveys, actions and strategies on systematically improving quality of care and promoting patient-centered care according to patient-reported experience measures have not yet been fully undertaken [67]. Considering the positive correlation between the patient experience and clinical outcomes, it needs to be considered as a tool to refine the quality of care and enhance the implementation of the concept of patient-centeredness [18, 64, 68]. In a recent study evaluating key drivers of the patient experience in pediatric population with heart disorders, cheerfulness during practice, the cohesiveness of staff, and explanation of problems and conditions from the providers were identified as predictive of overall satisfaction [69]. Furthermore, it has also been reported in a study evaluating interview narratives who had been hospitalized that, medication management, physical comfort, and emotional security were what matter most [70].

### Strengths and weaknesses of the study

One of the strengths of this overview of reviews is that a comprehensive search was conducted for studies relating to the patient experience. We provided evidence from different perspectives of the patient-healthcare provider relationship and summarized ten themes about the patient experience. Providers working in healthcare settings treating patients with musculoskeletal disorders may find this overview of reviews beneficial to better understand patients’ perceptions when using healthcare services, value of effective interpersonal skills, and need to simplify the process of access to quality healthcare. A few limitations of this overview of reviews included the unfeasibility of performing a meta-analysis (due to the heterogeneity among the study designs and population of included reviews) and lack of analysis of overlapping between the reviews. This means that one original study might have been included in more than one review. As we did not review all different musculoskeletal disorders, it may not necessarily be applicable to all health settings.

### Unanswered questions and future research

Further investigation of the patient experience should focus on patients with neurological disorders or other chronic conditions that require intensive healthcare services. It is also worth discussing issues on cultural differences/impacts that are relevant/different in various countries or geographical regions. To bridge the evidence to clinical practice, it is healthcare providers’ responsibility to try to understand the patient experience when delivering services. When acknowledging the relational and functional aspects of patient experience, healthcare providers would value the importance of communication and strive to comprehend what truly matters to their patients, which could be their individual information needs, preferences of treatment, or expectations of a supportive healthcare environment. Collecting patients’ feedbacks will assist healthcare providers better evaluate their services and ensure the voices of service users are heard [71].

## Conclusion

Patient experience alongside safety and clinical effectiveness serve as the three pillars that enhance quality of healthcare and influence patients’ perspectives when receiving healthcare services. In healthcare settings, which currently treat musculoskeletal conditions, efforts on measuring and capturing patient experience could help guide improvement in healthcare providers’ interpersonal aspects, and patient’s expectations on how healthcare should be delivered. This overview of reviews identified constructs regarding patient experience of healthcare providers and health services and proposed ways to enhance healthcare experience of patients with musculoskeletal disorders. By adjusting healthcare providers’ professional attitudes and behaviors when interacting with patients, as well as changing environmental factors in healthcare facilities, an improvement in patient adherence to medical advice and regimens promoting health and well-being would be reasonably expected. Our findings suggested that healthcare providers understand the importance of patient information needs and expectations via effective communication. It is also recommended that patients be treated individually with personalized intervention plans in a supportive, comforting environment.

## Supplementary information

**Additional file 1.** Search Strategy Report.

**Additional file 2.** List of excluded studies with reasons (total 197 reviews).

## Data Availability

all data generated or analyzed during this study are included in this published article and its supplementary information files.

## Abbreviations

AMSTAR-2: A MeaSurement Tool to Assess systematic Reviews
MSK: Musculoskeletal
PREMs: Patient-reported experience measures
PRISMA: Preferred Reporting Items for Systematic Reviews and Meta-Analyses
PROSPERO: Prospective Register of Systematic Reviews
