# Supplementary material for: Patients’ perceptions with musculoskeletal disorders regarding their experience with healthcare providers and health services: an overview of reviews
Source: Arch Physiother. 2020 Sep 24;10:17. doi: 10.1186/s40945-020-00088-6 (PMC7517681; doi:10.1186/s40945-020-00088-6)
Supplement: Supplementary file 1 — Additional file 1. Search Strategy Report. [file 40945_2020_88_MOESM1_ESM.docx]

**Appendix 1**

Search Strategy Report

Topic: Patient Experience Overview of Reviews

Searcher: Leila Ledbetter

Date: May 24, 2019

Updated search: December 20, 2019 (†indicated updated search results)

Database (including vendor/platform): PubMed (Legacy)

| Set # |  | Results |
| --- | --- | --- |
| 1  Patient experience | "Patient experience"[tw] OR "patients experience"[tw] OR "patients' experience"[tw] OR "patient's experience"[tw] OR "Patient satisfaction"[tw] OR "patients satisfaction"[tw] OR "patients' satisfaction"[tw] OR "patient's satisfaction"[tw] OR "Patient perception"[tw] OR "patients perception"[tw] OR "patients' perception"[tw] OR "patient's perception"[tw] OR "Patient perceived"[tw] OR "patients perceive"[tw] OR "patients' perceive"[tw] OR "patients perceived"[tw] OR "patients' perceived"[tw] OR "patient's perceived"[tw] OR "patient preferences"[tiab] OR "patients preferences"[tiab] OR "patients' preferences"[tiab] OR "patient's preferences"[tiab] OR "patient centered"[tiab] OR "patient centeredness"[tiab] OR "patient engagement"[tiab] OR "patient-clinician"[tiab] OR "clinician-patient"[tiab] OR "patient-doctor"[tiab] OR "doctor-patient"[tiab] OR "physician-patient"[tiab] OR "patient-physician"[tiab] OR "patient-provider"[tiab] OR ((patient[tiab] OR patients[tiab] OR patients'[tiab] OR "patient's"[tiab]) AND ("interpersonal treatment"[tiab] OR "physician discussion"[tiab] OR "trust in physician"[tiab] OR empathy[tiab] OR compassion[tiab] OR respect[tiab] OR "shared decision making"[tw] OR "therapeutic alliance"[tw] OR "participation in decisions"[tw] OR autonomy[tiab] OR caring[tiab] OR kindness[tiab] OR dignity[tiab] OR honesty[tiab] OR participation[tiab] OR "right to decide"[tiab] OR "physical comfort"[tiab] OR involvement[tiab] OR "emotional support"[tw] OR "clinical interaction"[tw] OR "continuity of care"[tw] OR "organizational culture"[tw] OR "smooth transition"[tw])) | 480,624  498,932† |
| 2  MSK | "Musculoskeletal Diseases"[Mesh] OR "Musculoskeletal System"[Mesh] OR "Manipulation, Orthopedic"[Mesh] OR "Orthopedics"[Mesh] OR "Orthopedic Procedures"[Mesh] OR "Osteopathic Medicine"[Mesh] OR osteopath*[tiab] OR Musculoskelet*[tiab] OR MSK[tiab] OR orthopedic*[tiab] OR "Osteoarthritis"[Mesh] OR osteoarthritis[tiab] OR cartilage[tiab] OR cartilages[tiab] OR "Fibromyalgia"[Mesh] OR fibrocartilage[tiab] OR Fibrocartilages[tiab] OR fascia*[tiab] OR ligament*[tiab] OR "connective tissue"[tiab] OR "connective tissues"[tiab] OR muscl*[tiab] OR bone[tiab] OR bones[tiab] OR joint[tiab] OR joints[tiab] OR tendon[tiab] OR tendons[tiab] OR menis*[tiab] OR fibros*[tiab] OR pulpos*[tiab] OR Sesamoid*[tiab] OR synov*[tiab] OR spine[tiab] OR spinal[tiab] OR "Back Pain"[Mesh] OR back[tiab] OR lumbar[tiab] OR lumbo*[tiab] OR verteb*[tiab] OR interverteb*[tiab] OR disc[tiab] OR discs[tiab] OR cervic*[tiab] OR thoracic[tiab] OR sacroli*[tiab] OR pelvi*[tiab] OR "Neck Pain"[Mesh] OR neck[tiab] OR arm[tiab] OR arms[tiab] OR elbow[tiab] OR elbows[tiab] OR shoulder[tiab] OR shoulders[tiab] OR wrist[tiab] OR wrists[tiab] hand[tiab] OR hands[tiab] OR carpal[tiab] OR metacarp*[tiab] OR palmar*[tiab] OR interphal*[tiab] OR phalange*[tiab] OR finger[tiab] OR fingers[tiab] OR knuckle[tiab] OR knuckles[tiab] OR rotator[tiab] OR sacroiliac[tiab] OR rib[tiab] OR ribs[tiab] OR flank[tiab] OR flanks[tiab] OR diaphragm*[tiab] OR clavic*[tiab] OR sternum[tiab] OR sternal[tiab] OR chest[tiab] OR hip[tiab] OR hips[tiab] OR leg[tiab] OR legs[tiab] OR knee[tiab] OR knees[tiab] OR ankle[tiab] OR ankles[tiab] OR foot[tiab] OR feet[tiab] OR tarsal[tiab] OR metatars*[tiab] OR plantar*[tiab] OR toe[tiab] OR toes[tiab] OR myofasci*[tiab] OR tenon*[tiab] OR face[tiab] OR facial[tiab] OR jaw[tiab] OR jaws[tiab] OR mandib*[tiab] OR maxill*[tiab] OR skull[tiab] OR skulls[tiab] OR cranium[tiab] OR cranial[tiab] OR cranio*[tiab] OR "non-headache"[tiab] OR nonheadache[tiab] OR osteoarthritis[tw] OR Arthritis[tw] OR Spondylitis[tw] OR Spondylarthritis[tw] OR Spondylarthropathies[tw] OR Osteoporosis[tw] OR "Back Pain"[Mesh] OR "Low Back Pain"[Mesh] OR "lower back"[tw] OR "low back"[tw] OR "Physical Therapy Modalities"[Mesh] OR "Physical Therapists"[Mesh] OR "Physical Therapists"[tw] OR "Physical Therapist"[tw] OR physiotherapy[tw] OR physiotherapists[tw] OR physiotherapist[tw] | 1,938,346  1,997,937† |
| 3  Reviews | (systematic review [ti] OR meta-analysis [pt] OR meta-analysis [ti] OR systematic literature review [ti] OR systematic review [tw] OR (systematic review [tiab] AND review [pt]) OR meta synthesis [ti] OR meta-analy*[ti] OR rapid review [tw] OR umbrella review [tw] OR scoping review[tw] OR practice guideline [pt] OR cochrane database syst rev [ta] OR jbi database system rev implement rep [ta] OR (clinical guideline [tw] AND management [tw]) OR ((systematic [tw] OR systematically [tw] OR study selection [tw] OR inclusion criteri* [tw] OR exclusion criteri* [tw]) AND (survey [tiab] OR surveys [tiab] OR overview* [tw] OR review [tiab] OR reviews [tiab] OR search* [tw] OR handsearch [tw] OR analysis [ti]))) AND (literature [tiab] OR articles [tiab] OR publications [tiab] OR publication [tiab] OR bibliography [tiab] OR bibliographies [tiab] OR published [tiab] OR pooled data [tw] OR unpublished [tw] OR citation [tw] OR citations [tw] OR database [tiab] OR internet [tiab] OR references [tw] OR scales [tw] OR papers [tw] OR datasets [tw] OR trials [tiab] OR meta-analy* [tw] OR (clinical [tiab] AND studies [tiab])) NOT (letter [pt] OR newspaper article [pt]) | 264,290  281,671† |
| 5 | 1 AND 2 AND 3 | 2,299  2,458† |
| 6 | 5 AND ("2019/05/01"[PDAT] : "3000/12/31"[PDAT]) | 192 |

Database (including vendor/platform): Embase (Elsevier)

| Set # |  | Results |
| --- | --- | --- |
| 1  Patient experience | 'Patient experience':ti,ab,kw OR 'patients experience':ti,ab,kw OR 'Patient satisfaction':ti,ab,kw OR 'patients satisfaction':ti,ab,kw OR 'Patient perception':ti,ab,kw OR 'patients perception':ti,ab,kw OR 'Patient perceived':ti,ab,kw OR 'patients perceive':ti,ab,kw OR 'patients perceived':ti,ab,kw OR 'patient preferences':ti,ab OR 'patients preferences':ti,ab OR 'patient centered':ti,ab OR 'patient centeredness':ti,ab OR 'patient engagement':ti,ab OR 'patient-clinician':ti,ab OR 'clinician-patient':ti,ab OR 'patient-doctor':ti,ab OR 'doctor-patient':ti,ab OR 'physician-patient':ti,ab OR 'patient-physician':ti,ab OR 'patient-provider':ti,ab OR ((patient:ti,ab OR patients:ti,ab OR patients:ti,ab) AND ('interpersonal treatment':ti,ab OR 'physician discussion':ti,ab OR 'trust in physician':ti,ab OR empathy:ti,ab OR compassion:ti,ab OR respect:ti,ab OR 'shared decision making':ti,ab,kw OR 'therapeutic alliance':ti,ab,kw OR 'participation in decisions':ti,ab,kw OR autonomy:ti,ab OR caring:ti,ab OR kindness:ti,ab OR dignity:ti,ab OR honesty:ti,ab OR participation:ti,ab OR 'right to decide':ti,ab OR 'physical comfort':ti,ab OR involvement:ti,ab OR 'emotional support':ti,ab,kw OR 'clinical interaction':ti,ab,kw OR 'continuity of care':ti,ab,kw OR 'organizational culture':ti,ab,kw OR 'smooth transition':ti,ab,kw)) | 654,553  686,761† |
| 2  MSK | 'musculoskeletal disease'/exp OR 'musculoskeletal system'/exp OR 'orthopedic manipulation'/exp OR 'orthopedics'/exp OR 'orthopedic procedures'/exp OR 'osteopathic medicine'/exp OR osteopath*:ti,ab OR Musculoskelet*:ti,ab OR MSK:ti,ab OR orthopedic*:ti,ab OR 'osteoarthritis'/exp OR osteoarthritis:ti,ab OR cartilage:ti,ab OR cartilages:ti,ab OR 'Fibromyalgia'/exp OR fibrocartilage:ti,ab OR Fibrocartilages:ti,ab OR fascia*:ti,ab OR ligament*:ti,ab OR 'connective tissue':ti,ab OR 'connective tissues':ti,ab OR muscl*:ti,ab OR bone:ti,ab OR bones:ti,ab OR joint:ti,ab OR joints:ti,ab OR tendon:ti,ab OR tendons:ti,ab OR menis*:ti,ab OR fibros*:ti,ab OR pulpos*:ti,ab OR Sesamoid*:ti,ab OR synov*:ti,ab OR spine:ti,ab OR spinal:ti,ab OR back:ti,ab OR lumbar:ti,ab OR lumbo*:ti,ab OR verteb*:ti,ab OR interverteb*:ti,ab OR disc:ti,ab OR discs:ti,ab OR cervic*:ti,ab OR thoracic:ti,ab OR sacroli*:ti,ab OR pelvi*:ti,ab OR 'Neck Pain'/exp OR neck:ti,ab OR arm:ti,ab OR arms:ti,ab OR elbow:ti,ab OR elbows:ti,ab OR shoulder:ti,ab OR shoulders:ti,ab OR wrist:ti,ab OR wrists:ti,ab hand:ti,ab OR hands:ti,ab OR carpal:ti,ab OR metacarp*:ti,ab OR palmar*:ti,ab OR interphal*:ti,ab OR phalange*:ti,ab OR finger:ti,ab OR fingers:ti,ab OR knuckle:ti,ab OR knuckles:ti,ab OR rotator:ti,ab OR sacroiliac:ti,ab OR rib:ti,ab OR ribs:ti,ab OR flank:ti,ab OR flanks:ti,ab OR diaphragm*:ti,ab OR clavic*:ti,ab OR sternum:ti,ab OR sternal:ti,ab OR chest:ti,ab OR hip:ti,ab OR hips:ti,ab OR leg:ti,ab OR legs:ti,ab OR knee:ti,ab OR knees:ti,ab OR ankle:ti,ab OR ankles:ti,ab OR foot:ti,ab OR feet:ti,ab OR tarsal:ti,ab OR metatars*:ti,ab OR plantar*:ti,ab OR toe:ti,ab OR toes:ti,ab OR myofasci*:ti,ab OR tenon*:ti,ab OR face:ti,ab OR facial:ti,ab OR jaw:ti,ab OR jaws:ti,ab OR mandib*:ti,ab OR maxill*:ti,ab OR skull:ti,ab OR skulls:ti,ab OR cranium:ti,ab OR cranial:ti,ab OR cranio*:ti,ab OR 'non-headache':ti,ab OR nonheadache:ti,ab OR osteoarthritis:ti,ab,kw OR Arthritis:ti,ab,kw OR Spondylitis:ti,ab,kw OR Spondylarthritis:ti,ab,kw OR Spondylarthropathies:ti,ab,kw OR Osteoporosis:ti,ab,kw OR 'backache'/exp OR 'low back pain'/exp OR 'lower back':ti,ab,kw OR 'low back':ti,ab,kw OR 'Physical Therapy':ti,ab,kw OR 'physiotherapy'/exp OR 'physiotherapist'/exp OR 'Physical Therapists':ti,ab,kw OR 'Physical Therapist':ti,ab,kw OR physiotherapy:ti,ab,kw OR physiotherapists:ti,ab,kw OR physiotherapist:ti,ab,kw | 2,559,342  2,650,459† |
| 3  Reviews | ('systematic review':ti OR meta-analysis:pt OR meta-analysis:ti OR 'systematic literature review':ti OR 'systematic review':ti,ab,kw OR ('systematic review':ti,ab AND review:pt) OR 'meta synthesis':ti OR meta-analysis:ti OR meta-analyses:ti OR 'rapid review':ti,ab,kw OR 'umbrella review':ti,ab,kw OR 'scoping review':ti,ab,kw OR 'practice guideline':pt OR ('clinical guideline':ti,ab,kw AND management:ti,ab,kw) OR ((systematic:ti,ab,kw OR systematically:ti,ab,kw OR 'study selection':ti,ab,kw OR 'inclusion criteria':ti,ab,kw OR 'inclusion criterias':ti,ab,kw OR 'inclusion criterion':ti,ab,kw OR 'inclusion criterions':ti,ab,kw OR 'inclusion criterium':ti,ab,kw OR 'exclusion criteria':ti,ab,kw OR 'exclusion criterias':ti,ab,kw OR 'exclusion criterion':ti,ab,kw OR 'exclusion criterions':ti,ab,kw OR 'exclusion criterium':ti,ab,kw) AND (survey:ti,ab OR surveys:ti,ab OR overview:ti,ab,kw OR overviews:ti,ab,kw OR overviewed:ti,ab,kw or review:ti,ab OR reviews:ti,ab OR search:ti,ab,kw OR searches:ti,ab,kw OR searched:ti,ab,kw OR handsearch:ti,ab,kw OR analysis:ti))) AND (literature:ti,ab OR articles:ti,ab OR publications:ti,ab OR publication:ti,ab OR bibliography:ti,ab OR bibliographies:ti,ab OR published:ti,ab OR pooled data:ti,ab,kw OR unpublished:ti,ab,kw OR citation:ti,ab,kw OR citations:ti,ab,kw OR database:ti,ab OR internet:ti,ab OR references:ti,ab,kw OR scales:ti,ab,kw OR papers:ti,ab,kw OR datasets:ti,ab,kw OR trials:ti,ab OR meta-analysis:ti,ab,kw OR meta-analyses:ti,ab,kw OR (clinical:ti,ab AND studies:ti,ab) OR 'treatment outcome'/exp OR 'treatment outcome':ti,ab,kw) | 270,237  295,582† |
| 5 | 1 AND 2 AND 3 | 2,400  2,629† |
| 6 | 5 AND [1-5-2019]/sd | 347 |

Database (including vendor/platform): Scopus (Elsevier)

| Set # |  | Results |
| --- | --- | --- |
| 1  Patient experience | TITLE-ABS-KEY("Patient experience" OR "patients experience" OR "Patient satisfaction" OR "patients satisfaction" OR "Patient perception" OR "patients perception" OR "Patient perceived" OR "patients perceive" OR "patients perceived" OR "patient preferences" OR "patients preferences" OR "patient centered" OR "patient centeredness" OR "patient engagement" OR "patient-clinician" OR "clinician-patient" OR "patient-doctor" OR "doctor-patient" OR "physician-patient" OR "patient-physician" OR "patient-provider" OR ((patient OR patients OR patients) AND ("interpersonal treatment" OR "physician discussion" OR "trust in physician" OR empathy OR compassion OR respect OR "shared decision making" OR "therapeutic alliance" OR "participation in decisions" OR autonomy OR caring OR kindness OR dignity OR honesty OR participation OR "right to decide" OR "physical comfort" OR involvement OR "emotional support" OR "clinical interaction" OR "continuity of care" OR "organizational culture" OR "smooth transition"))) | 779,748  806,895† |
| 2  MSK | TITLE-ABS-KEY("musculoskeletal disease" OR "musculoskeletal system" OR "orthopedic manipulation" OR "orthopedics" OR "orthopedic procedures" OR "osteopathic medicine" OR osteopath* OR Musculoskelet* OR MSK OR orthopedic* OR "osteoarthritis" OR osteoarthritis OR cartilage OR cartilages OR "Fibromyalgia" OR fibrocartilage OR Fibrocartilages OR fascia* OR ligament* OR "connective tissue" OR "connective tissues" OR muscl* OR bone OR bones OR joint OR joints OR tendon OR tendons OR menis* OR fibros* OR pulpos* OR Sesamoid* OR synov* OR spine OR spinal OR back OR lumbar OR lumbo* OR verteb* OR interverteb* OR disc OR discs OR cervic* OR thoracic OR sacroli* OR pelvi* OR "Neck Pain" OR neck OR arm OR arms OR elbow OR elbows OR shoulder OR shoulders OR wrist OR wrists hand OR hands OR carpal OR metacarp* OR palmar* OR interphal* OR phalange* OR finger OR fingers OR knuckle OR knuckles OR rotator OR sacroiliac OR rib OR ribs OR flank OR flanks OR diaphragm* OR clavic* OR sternum OR sternal OR chest OR hip OR hips OR leg OR legs OR knee OR knees OR ankle OR ankles OR foot OR feet OR tarsal OR metatars* OR plantar* OR toe OR toes OR myofasci* OR tenon* OR face OR facial OR jaw OR jaws OR mandib* OR maxill* OR skull OR skulls OR cranium OR cranial OR cranio* OR "non-headache" OR nonheadache OR osteoarthritis OR Arthritis OR Spondylitis OR Spondylarthritis OR Spondylarthropathies OR Osteoporosis OR "backache" OR "low back pain" OR "lower back" OR "low back" OR "Physical Therapy" OR "physiotherapy" OR "physiotherapist" OR "Physical Therapists" OR "Physical Therapist" OR physiotherapy OR physiotherapists OR physiotherapist) | 1,598,693  1,645,681† |
| 3  Reviews | TITLE-ABS ( ( "systematic review" OR meta-analysis OR meta-analyses OR meta-analytic OR "systematic literature review" OR "systematic review" OR ( "systematic review" AND review ) OR "meta synthesis" OR meta-analyses OR meta-analytic OR "rapid review" OR "umbrella review" OR "scoping review" OR "practice guideline" OR ( "clinical guideline" AND management ) OR ( ( systematic OR systematically OR "study selection" OR "inclusion criteria" OR "inclusion criterias" OR "inclusion criterion" OR "inclusion criterions" OR "inclusion criterium" OR "exclusion criteria" OR "exclusion criterias" OR "exclusion criterion" OR "exclusion criterions" OR "exclusion criterium" ) AND ( survey OR surveys OR overview OR overviews OR overviewed OR review OR reviews OR search OR searched OR handsearch OR analysis ) ) ) AND ( literature OR articles OR publications OR publication OR bibliography OR bibliographies OR published OR "pooled data" OR unpublished OR citation OR citations OR database OR internet OR references OR scales OR papers OR datasets OR trials OR meta-analysis OR meta-analyses OR ( clinical AND studies ) OR "treatment outcome" OR "treatment outcomes" ) ) | 467,721  503,107† |
| 5 | 1 AND 2 AND 3 | 2,527  2,696† |
| 6 | 5 AND ( LIMIT-TO ( PUBYEAR , 2019 ) ) | 277 |

Database (including vendor/platform): CINAHL Complete (EbscoHost)

| Set # |  | Results |
| --- | --- | --- |
| 1  Patient experience | TI ("Patient experience" OR "patients experience" OR "Patient satisfaction" OR "patients satisfaction" OR "Patient perception" OR "patients perception" OR "Patient perceived" OR "patients perceive" OR "patients perceived" OR "patient preferences" OR "patients preferences" OR "patient centered" OR "patient centeredness" OR "patient engagement" OR "patient-clinician" OR "clinician-patient" OR "patient-doctor" OR "doctor-patient" OR "physician-patient" OR "patient-physician" OR "patient-provider" OR ((patient OR patients OR patients) AND ("interpersonal treatment" OR "physician discussion" OR "trust in physician" OR empathy OR compassion OR respect OR "shared decision making" OR "therapeutic alliance" OR "participation in decisions" OR autonomy OR caring OR kindness OR dignity OR honesty OR participation OR "right to decide" OR "physical comfort" OR involvement OR "emotional support" OR "clinical interaction" OR "continuity of care" OR "organizational culture" OR "smooth transition"))) OR AB ("Patient experience" OR "patients experience" OR "Patient satisfaction" OR "patients satisfaction" OR "Patient perception" OR "patients perception" OR "Patient perceived" OR "patients perceive" OR "patients perceived" OR "patient preferences" OR "patients preferences" OR "patient centered" OR "patient centeredness" OR "patient engagement" OR "patient-clinician" OR "clinician-patient" OR "patient-doctor" OR "doctor-patient" OR "physician-patient" OR "patient-physician" OR "patient-provider" OR ((patient OR patients OR patients) AND ("interpersonal treatment" OR "physician discussion" OR "trust in physician" OR empathy OR compassion OR respect OR "shared decision making" OR "therapeutic alliance" OR "participation in decisions" OR autonomy OR caring OR kindness OR dignity OR honesty OR participation OR "right to decide" OR "physical comfort" OR involvement OR "emotional support" OR "clinical interaction" OR "continuity of care" OR "organizational culture" OR "smooth transition"))) | 122,048  130,834† |
| 2  MSK | (MH "Musculoskeletal Diseases+") OR (MH "Musculoskeletal System+") OR (MH "Orthopedics") OR (MH "Physical Therapy+") OR (MH "Physical Therapists")  OR TI ("musculoskeletal disease" OR "musculoskeletal system" OR "orthopedic manipulation" OR "orthopedics" OR "orthopedic procedures" OR "osteopathic medicine" OR osteopath* OR Musculoskelet* OR MSK OR orthopedic* OR "osteoarthritis" OR osteoarthritis OR cartilage OR cartilages OR "Fibromyalgia" OR fibrocartilage OR Fibrocartilages OR fascia* OR ligament* OR "connective tissue" OR "connective tissues" OR muscl* OR bone OR bones OR joint OR joints OR tendon OR tendons OR menis* OR fibros* OR pulpos* OR Sesamoid* OR synov* OR spine OR spinal OR back OR lumbar OR lumbo* OR verteb* OR interverteb* OR disc OR discs OR cervic* OR thoracic OR sacroli* OR pelvi* OR "Neck Pain" OR neck OR arm OR arms OR elbow OR elbows OR shoulder OR shoulders OR wrist OR wrists hand OR hands OR carpal OR metacarp* OR palmar* OR interphal* OR phalange* OR finger OR fingers OR knuckle OR knuckles OR rotator OR sacroiliac OR rib OR ribs OR flank OR flanks OR diaphragm* OR clavic* OR sternum OR sternal OR chest OR hip OR hips OR leg OR legs OR knee OR knees OR ankle OR ankles OR foot OR feet OR tarsal OR metatars* OR plantar* OR toe OR toes OR myofasci* OR tenon* OR face OR facial OR jaw OR jaws OR mandib* OR maxill* OR skull OR skulls OR cranium OR cranial OR cranio* OR "non-headache" OR nonheadache OR osteoarthritis OR Arthritis OR Spondylitis OR Spondylarthritis OR Spondylarthropathies OR Osteoporosis OR "backache" OR "low back pain" OR "lower back" OR "low back" OR "Physical Therapy" OR "physiotherapy" OR "physiotherapist" OR "Physical Therapists" OR "Physical Therapist" OR physiotherapy OR physiotherapists OR physiotherapist) OR AB ("musculoskeletal disease" OR "musculoskeletal system" OR "orthopedic manipulation" OR "orthopedics" OR "orthopedic procedures" OR "osteopathic medicine" OR osteopath* OR Musculoskelet* OR MSK OR orthopedic* OR "osteoarthritis" OR osteoarthritis OR cartilage OR cartilages OR "Fibromyalgia" OR fibrocartilage OR Fibrocartilages OR fascia* OR ligament* OR "connective tissue" OR "connective tissues" OR muscl* OR bone OR bones OR joint OR joints OR tendon OR tendons OR menis* OR fibros* OR pulpos* OR Sesamoid* OR synov* OR spine OR spinal OR back OR lumbar OR lumbo* OR verteb* OR interverteb* OR disc OR discs OR cervic* OR thoracic OR sacroli* OR pelvi* OR "Neck Pain" OR neck OR arm OR arms OR elbow OR elbows OR shoulder OR shoulders OR wrist OR wrists hand OR hands OR carpal OR metacarp* OR palmar* OR interphal* OR phalange* OR finger OR fingers OR knuckle OR knuckles OR rotator OR sacroiliac OR rib OR ribs OR flank OR flanks OR diaphragm* OR clavic* OR sternum OR sternal OR chest OR hip OR hips OR leg OR legs OR knee OR knees OR ankle OR ankles OR foot OR feet OR tarsal OR metatars* OR plantar* OR toe OR toes OR myofasci* OR tenon* OR face OR facial OR jaw OR jaws OR mandib* OR maxill* OR skull OR skulls OR cranium OR cranial OR cranio* OR "non-headache" OR nonheadache OR osteoarthritis OR Arthritis OR Spondylitis OR Spondylarthritis OR Spondylarthropathies OR Osteoporosis OR "backache" OR "low back pain" OR "lower back" OR "low back" OR "Physical Therapy" OR "physiotherapy" OR "physiotherapist" OR "Physical Therapists" OR "Physical Therapist" OR physiotherapy OR physiotherapists OR physiotherapist) | 1,016,905  1,065,905† |
|  | 1 AND 2 | 33,571  36,186† |
| 3  Reviews | **Limiters** - Publication Type: Meta Analysis, Meta Synthesis, Review, Systematic Review | 2,887  2,924† |
| 4 | 3 AND Published Date: 20190501- | 0 |
