# Supplementary material for: Patients’ perceptions with musculoskeletal disorders regarding their experience with healthcare providers and health services: an overview of reviews
Source: Arch Physiother. 2020 Sep 24;10:17. doi: 10.1186/s40945-020-00088-6 (PMC7517681; doi:10.1186/s40945-020-00088-6)
Supplement: Supplementary file 2 — Additional file 2. List of excluded studies with reasons (total 197 reviews). [file 40945_2020_88_MOESM2_ESM.docx]

**Appendix 2**

List of excluded studies with reasons (total 197 reviews)

| Did not investigate patient experience outcomes (n = 157) |
| --- |
| Abdallah, F.W., C. Madjdpour, and R. Brull, Is sciatic nerve block advantageous when combined with femoral nerve block for postoperative analgesia following total knee arthroplasty? a meta-analysis. Canadian journal of anaesthesia = Journal canadien d'anesthesie, 2016. 63(5): p. 552-68.  Adam, J.A., et al., Patient decision aids in joint replacement surgery: a literature review and an opinion survey of consultant orthopaedic surgeons. Annals of the Royal College of Surgeons of England, 2008. 90(3): p. 198-207.  Adams, J., et al., Proximal interphalangeal joint replacement in patients with arthritis of the hand: A meta-analysis. Journal of Bone and Joint Surgery - Series B, 2012. 94 B(10): p. 1305-1312.  Adie, S., J.M. Naylor, and I.A. Harris, Cryotherapy after total knee arthroplasty a systematic review and meta-analysis of randomized controlled trials. The Journal of arthroplasty, 2010. 25(5): p. 709-15.  Agarwalla, A., et al., Timeline for Maximal Subjective Outcome Improvement After Anterior Cruciate Ligament Reconstruction. The American journal of sports medicine, 2018: p. 363546518803365.  Agnihotry, A., et al., Resorbable versus titanium plates for orthognathic surgery. The Cochrane database of systematic reviews, 2017. 10: p. Cd006204.  Akram, J. and S.H. Matzen, Rectus abdominis diastasis. Journal of Plastic Surgery and Hand Surgery, 2014. 48(3): p. 163-169.  Aksoy, C.C., et al., The effectiveness of Nintendo Wii® exercises in rehabilitation following knee surgery. Fizyoterapi Rehabilitasyon, 2017. 28(2): p. S88-S89.  Akula, M., et al., Does the morbidity & mortality justify lumbar fusion surgery in older adults? Global Spine Journal, 2018. 8(1): p. 252S-253S.  Al-Qubaeissy, K.Y., et al., The effectiveness of hydrotherapy in the management of rheumatoid arthritis: a systematic review. Musculoskeletal care, 2013. 11(1): p. 3-18.  Alderman, A.K. and K.C. Chung, Measuring Outcomes in Hand Surgery. Clinics in Plastic Surgery, 2008. 35(2): p. 239-250.  Althof, J.E. and B.D. Beasley, Psychosocial management of the foot and ankle surgery patient. Clinics in Podiatric Medicine & Surgery, 2003. 20(2): p. 199-211.  Aman, M.M., et al., Evidence-Based Non-Pharmacological Therapies for Fibromyalgia. Current Pain and Headache Reports, 2018. 22(5).  Ammer, K., Physiotherapy in seronegative spondylarthropathies - A systematic review. European Journal of Physical Medicine and Rehabilitation, 1997. 7(4): p. 114-119.  Andersen, J.C., Is immediate imaging important in managing low back pain? Journal of athletic training, 2011. 46(1): p. 99-102.  Andriolo, L., et al., Revision anterior cruciate ligament reconstruction: clinical outcome and evidence for return to sport. Knee Surgery, Sports Traumatology, Arthroscopy, 2015. 23(10): p. 2825-2845.  Anichini, G., et al., Lumbar Endoscopic Microdiscectomy: Where Are We Now? An Updated Literature Review Focused on Clinical Outcome, Complications, and Rate of Recurrence. BioMed Research International, 2015. 2015: p. 1-14.  Ardern, C.L., et al., Fifty-five per cent return to competitive sport following anterior cruciate ligament reconstruction surgery: an updated systematic review and meta-analysis including aspects of physical functioning and contextual factors. British journal of sports medicine, 2014. 48(21): p. 1543-52.  Argueta-Bernal, G., Behavioral approaches for chronic low back pain. Seminars in Pain Medicine, 2004. 2(3): p. 197-202.  Arshi, A., et al., Can Biologic Augmentation Improve Clinical Outcomes Following Microfracture for Symptomatic Cartilage Defects of the Knee? A Systematic Review. Cartilage, 2018. 9(2): p. 146-155.  Ashbrook, J., et al., 43 Management of acute low back pain in the ED: a systematic review. Emergency medicine journal : EMJ, 2017. 34(12): p. A889.  Asif, M., et al., Patient and caregiver experiences on care transitions for adults with a hip fracture: a scoping review. Disability and rehabilitation, 2019: p. 1-10.  Askari, M., et al., Assessing quality of care of elderly patients using the ACOVE quality indicator set: a systematic review. PloS one, 2011. 6(12): p. e28631.  Aujla, R.S., et al., Unconstrained metacarpophalangeal joint arthroplasties: a systematic review. The bone & joint journal, 2017. 99-b(1): p. 100-106.  Aydin, D., et al., No major effects of preoperative education in patients undergoing hip or knee replacement--a systematic review. Danish medical journal, 2015. 62(7).  Babatunde, F., J. MacDermid, and N. MacIntyre, Characteristics of therapeutic alliance in musculoskeletal physiotherapy and occupational therapy practice: a scoping review of the literature. BMC health services research, 2017. 17(1): p. 375.  Baez, S., M.C. Hoch, and J.M. Hoch, Evaluation of Cognitive Behavioral Interventions and Psychoeducation Implemented by Rehabilitation Specialists to Treat Fear-Avoidance Beliefs in Patients With Low Back Pain: A Systematic Review. Archives of physical medicine and rehabilitation, 2018. 99(11): p. 2287-2298.  Ballesteros, V., et al., Effectiveness of the microendoscopic discectomy versus open discectomy for the treatment of symptomatic lumbar disc herniation: Meta-analysis. Global Spine Journal, 2017. 7(2): p. 304S-305S.  Baltes, T., et al., Surgical treatment for midportion Achilles tendinopathy: a systematic review. Knee Surgery, Sports Traumatology, Arthroscopy, 2017. 25(6): p. 1817-1838.  Barker, K.L., F. Toye, and C.J. Lowe, A qualitative systematic review of patients' experience of osteoporosis using meta-ethnography. Archives of osteoporosis, 2016. 11(1): p. 33.  Barlow, T., et al., Patients' decision making in total knee arthroplasty: a systematic review of qualitative research. Bone & joint research, 2015. 4(10): p. 163-9.  Barnsley, L. and R. Page, Are Hip Precautions Necessary Post Total Hip Arthroplasty? A Systematic Review. Geriatric orthopaedic surgery & rehabilitation, 2015. 6(3): p. 230-5.  Barton, J., et al., Patient treatment goals in rheumatoid arthritis: Results of focus groups among rheumatologists, English and Spanish-speaking patients. Arthritis and Rheumatology, 2014. 66: p. S1055-S1056.  Bedi, A., et al., The management of labral tears and femoroacetabular impingement of the hip in the young, active patient. Arthroscopy : the journal of arthroscopic & related surgery : official publication of the Arthroscopy Association of North America and the International Arthroscopy Association, 2008. 24(10): p. 1135-45.  Blau, W.S., et al., Evaluation and treatment of chronic lumbar facet joint pain: clinical guideline of the unc pain management center. Regional Anesthesia, 1997. 22(2 SUPPL.): p. 103.  Blom, A.W., et al., Programme Grants for Applied Research. 2016, NIHR Journals Library Copyright (c) Queen's Printer and Controller of HMSO 2016. This work was produced by Blom et al. under the terms of a commissioning contract issued by the Secretary of State for Health. This issue may be freely reproduced for the purposes of private research and study and extracts (or indeed, the full report) may be included in professional journals provided that suitable acknowledgement is made and the reproduction is not associated with any form of advertising. Applications for commercial reproduction should be addressed to: NIHR Journals Library, National Institute for Health Research, Evaluation, Trials and Studies Coordinating Centre, Alpha House, University of Southampton Science Park, Southampton SO16 7NS, UK.: Southampton (UK).  Boehmer, K.R., et al., Patient capacity and constraints in the experience of chronic disease: a qualitative systematic review and thematic synthesis. BMC Family Practice, 2016. 17: p. 1-23.  Bogunovic, L., et al., Treatment of tibial eminence fractures: a systematic review. The journal of knee surgery, 2015. 28(3): p. 255-262.  Boissonnault, W., Clinical factors leading to physical therapists referring patients to physicians: A systematic review paper. Physiotherapy (United Kingdom), 2011. 97: p. eS145-eS146.  Borkhoff, C.M., et al., Reaching those most in need: A scoping review of interventions to improve health care quality for disadvantaged populations with osteoarthritis. Osteoarthritis and Cartilage, 2010. 18: p. S163.  Brambilla, L., et al., Lateral ankle ligament anatomic reconstruction for chronic ankle instability: Allograft or autograft? A systematic review. Foot and ankle surgery : official journal of the European Society of Foot and Ankle Surgeons, 2018.  Bream, E. and N. Black, What is the relationship between patients' and clinicians' reports of the outcomes of elective surgery? Journal of health services research & policy, 2009. 14(3): p. 174-82.  Breuil, V., C.H. Roux, and G.F. Carle, Pelvic fractures: epidemiology, consequences, and medical management. Current Opinion in Rheumatology, 2016. 28(4): p. 442-447.  Bright, P. and K. Hambly, What Is the Proportion of Studies Reporting Patient and Practitioner Satisfaction with Software Support Tools Used in the Management of Knee Pain and Is This Related to Sample Size, Effect Size, and Journal Impact Factor? Telemedicine journal and e-health : the official journal of the American Telemedicine Association, 2018. 24(8): p. 562-576.  Buecking, B., et al., Early orthogeriatric treatment of trauma in the elderly: A systematic review and metaanalysis. Deutsches Arzteblatt International, 2014. 110(15): p. 255-262.  Busija, L., et al., Systematic review showed measures of individual burden of osteoarthritis poorly capture the patient experience. Journal of clinical epidemiology, 2013. 66(8): p. 826-37.  Butts, S.C., et al., Reporting of Postoperative Pain Management Protocols in Randomized Clinical Trials of Mandibular Fracture Repair: A Systematic Review. JAMA facial plastic surgery, 2015. 17(6): p. 440-8.  Cabrera Martimbianco, A.L., et al., Effectiveness and safety of cryotherapy after arthroscopic anterior cruciate ligament reconstruction. A systematic review of the literature. Physical Therapy in Sport, 2014. 15(4): p. 261-268.  Candelas, G., et al., Benefit of health education by a training nurse in patients with axial and/or peripheral psoriatic arthritis: A systematic literature review. Rheumatology international, 2016. 36(11): p. 1493-1506.  Carere, A. and R. Orr, The impact of hydrotherapy on a patient’s perceived well-being: a critical review of the literature. Physical Therapy Reviews, 2016. 21(2): p. 91-101.  Chahal, J., et al., Outcomes of osteochondral allograft transplantation in the knee. Arthroscopy - Journal of Arthroscopic and Related Surgery, 2013. 29(3): p. 575-588.  Chahla, J., et al., Osteochondral Allograft Transplantation in the Patellofemoral Joint: A Systematic Review. The American journal of sports medicine, 2018: p. 363546518814236.  Cheriyan, T., et al., Association between compensation status and outcomes in spine surgery: a meta-analysis of 31 studies. Spine Journal, 2015. 15(12): p. 2564-2573.  Cook, E., et al., Meta-analysis of first metatarsophalangeal joint implant arthroplasty. The Journal of foot and ankle surgery : official publication of the American College of Foot and Ankle Surgeons, 2009. 48(2): p. 180-90.  Côté, P., et al., Non-pharmacological management of persistent headaches associated with neck pain: A clinical practice guideline from the Ontario protocol for traffic injury management (OPTIMa) collaboration. European Journal of Pain (United Kingdom), 2019.  Coylewright, M., et al., Impact of sociodemographic patient characteristics on the efficacy of decision AIDS: a patient-level meta-analysis of 7 randomized trials. Circulation. Cardiovascular quality and outcomes, 2014. 7(3): p. 360-7.  Cozzi, A.L., et al., Kinesiophobia After Anterior Cruciate Ligament Reconstruction in Physically Active Individuals. Journal of Sport Rehabilitation, 2015. 24(4): p. 434-439.  Cucchi, D., et al., Early combined arthroscopic treatment for simultaneous ruptures of the patellar tendon and the anterior cruciate ligament leads to good radiological results and patient satisfaction. Knee surgery, sports traumatology, arthroscopy : official journal of the ESSKA, 2018. 26(4): p. 1164-1173.  Cui, X.D., et al., Mid- to long-term results of total disc replacement for lumbar degenerative disc disease: a systematic review. Journal of orthopaedic surgery and research, 2018. 13(1): p. 326.  Culliton, S.E., et al., The relationship between expectations and satisfaction in patients undergoing primary total knee arthroplasty. The Journal of arthroplasty, 2012. 27(3): p. 490-2.  DeLong, J.M., K. Jiang, and J.P. Bradley, Posterior Instability of the Shoulder. American Journal of Sports Medicine, 2015. 43(7): p. 1805-1817.  Desmeules, F., et al., Advanced practice physiotherapy in patients with musculoskeletal disorders: a systematic review. BMC musculoskeletal disorders, 2012. 13: p. 107.  Dianne Liddle, S., J.H. Gracey, and G. David Baxter, Advice for the management of low back pain: A systematic review of randomised controlled trials. Manual Therapy, 2007. 12(4): p. 310-327.  Dilla, T., et al., Patients' Preferences for Rheumatoid Arthritis Treatments and their Participation in the Treatment Decision-Making Process. A Systematic Review of the Literature. Value in health : the journal of the International Society for Pharmacoeconomics and Outcomes Research, 2015. 18(7): p. A652.  Dower, A., et al., Surgical management of recurrent lumbar disc herniation and the role of fusion. Journal of Clinical Neuroscience, 2016. 23: p. 44-50.  Durand, C., et al., Patient preferences for disease modifying anti-rheumatic drug treatment of rheumatoid arthritis: A systematic review. Journal of Rheumatology, 2017. 44(6): p. 889.  Ellis, D.J., et al., The Relationship between Preoperative Expectations and the Short-Term Postoperative Satisfaction and Functional Outcome in Lumbar Spine Surgery: A Systematic Review. Global Spine Journal, 2015. 5(5): p. 436-451.  Eschalier, B. and E. Coudeyre, The role of patient education before total knee arthroplasty. Lettre de Medecine Physique et de Readaptation, 2013. 29(3): p. 110-118.  Froud, R., et al., A systematic review and meta-synthesis of the impact of low back pain on people's lives. BMC Musculoskeletal Disorders, 2014. 15(1).  Gossec, L., et al., Reporting of patient-perceived impact of rheumatoid arthritis and axial spondyloarthritis over 10 years: a systematic literature review. Rheumatology (Oxford, England), 2014. 53(7): p. 1274-81.  Gross, A., et al., Patient education for neck pain. Cochrane Database of Systematic Reviews, 2012(3): p. N.PAG-N.PAG.  Gunaratne, R., et al., Patient Dissatisfaction Following Total Knee Arthroplasty: A Systematic Review of the Literature. The Journal of arthroplasty, 2017. 32(12): p. 3854-3860.  Haanstra, T.M., et al., Systematic review: do patient expectations influence treatment outcomes in total knee and total hip arthroplasty? Health and quality of life outcomes, 2012. 10: p. 152.  Hall, A.M., et al., The influence of the therapist-patient relationship on treatment outcome in physical rehabilitation: A systematic review. Physical Therapy, 2010. 90(8): p. 1099-1110.  Hammett, T., et al., Changes in Physical Activity After Total Hip or Knee Arthroplasty: A Systematic Review and Meta-Analysis of Six- and Twelve-Month Outcomes. Arthritis Care and Research, 2018. 70(6): p. 892-901.  Hoorntje, A., et al., Most patients return to work and sports after total hip arthroplasty-a systematic review and metaanalysis. HIP International, 2018. 28: p. 66.  Hoorntje, A., et al., The Effect of Total Hip Arthroplasty on Sports and Work Participation: A Systematic Review and Meta-Analysis. Sports medicine (Auckland, N.Z.), 2018. 48(7): p. 1695-1726.  Horner, N.S., et al., Indications and outcomes of shoulder arthroscopy after shoulder arthroplasty. Journal of shoulder and elbow surgery, 2016. 25(3): p. 510-8.  Houston, M.N., J.M. Hoch, and M.C. Hoch, Patient-Reported Outcome Measures in Individuals With Chronic Ankle Instability: A Systematic Review. Journal of athletic training, 2015. 50(10): p. 1019-33.  Hoving, J.L., et al., Work participation and arthritis: a systematic overview of challenges, adaptations and opportunities for interventions. Rheumatology (Oxford, England), 2013. 52(7): p. 1254-64.  Hui-Hui, S., et al., The efficacy and safety of using cooled radiofrequency in treating chronic sacroiliac joint pain: A PRISMA-compliant meta-analysis. Medicine, 2018. 97(6): p. 1-8.  Hulen, E., et al., Patient goals in rheumatoid arthritis care: A systematic review and qualitative synthesis. Musculoskeletal care, 2017. 15(4): p. 295-303.  Hurley, M., et al., Exercise interventions and patient beliefs for people with hip, knee or hip and knee osteoarthritis: A mixed methods review. Cochrane Database of Systematic Reviews, 2018. 2018(4).  Jain, S. and P.V. Giannoudis, Arthrodesis of the hip and conversion to total hip arthroplasty: a systematic review. The Journal of arthroplasty, 2013. 28(9): p. 1596-602.  Jauregui, J.J., et al., Conversion of a Surgically Arthrodesed Knee to a Total Knee Arthroplasty-Is it Worth it? A Meta-Analysis. The Journal of arthroplasty, 2016. 31(8): p. 1736-41.  Jones, C.A. and M.E. Suarez-Almazor, Patient Expectations and Total Knee Arthroplasty. Journal of Clinical Outcomes Management, 2017. 24(8): p. 364-370.  Jones, E.L., et al., A systematic review of patient reported outcomes and patient experience in enhanced recovery after orthopaedic surgery. Annals of the Royal College of Surgeons of England, 2014. 96(2): p. 89-94.  Joseph, C., et al., Musculoskeletal triage: a mixed methods study, integrating systematic review with expert and patient perspectives. Physiotherapy, 2014. 100(4): p. 277-89.  Kahlenberg, C.A., et al., Patient Satisfaction After Total Knee Replacement: A Systematic Review. HSS journal : the musculoskeletal journal of Hospital for Special Surgery, 2018. 14(2): p. 192-201.  Kahlenberg, C.A., et al., Patient Satisfaction Reporting for the Treatment of Femoroacetabular Impingement. Arthroscopy : the journal of arthroscopic & related surgery : official publication of the Arthroscopy Association of North America and the International Arthroscopy Association, 2016. 32(8): p. 1693-9.  Kahlenberg, C.A., et al., Patient Satisfaction Reporting After Total Hip Arthroplasty: A Systematic Review. Orthopedics, 2017. 40(3): p. e400-e404.  Karel, Y.H.J.M., et al., Effect of routine diagnostic imaging for patients with musculoskeletal disorders: A meta-analysis. European Journal of Internal Medicine, 2015. 26(8): p. 585-595.  Kathryn, C., et al., Perceived health information needs in inflammatory arthritis: A review of the literature. Internal Medicine Journal, 2016. 46: p. 11-12.  Kierkegaard, S., et al., Pain, activities of daily living and sport function at different time points after hip arthroscopy in patients with femoroacetabular impingement: a systematic review with meta-analysis. British journal of sports medicine, 2017. 51(7): p. 572-579.  Kinney, M., et al., The impact of therapeutic alliance in physical therapy for chronic musculoskeletal pain: A systematic review of the literature. Physiotherapy theory and practice, 2018: p. 1-13.  Kunutsor, S.K., et al., Health Care Needs and Support for Patients Undergoing Treatment for Prosthetic Joint Infection following Hip or Knee Arthroplasty: A Systematic Review. PloS one, 2017. 12(1): p. e0169068.  Ladermann, A., P.J. Denard, and S.S. Burkhart, Revision arthroscopic rotator cuff repair: systematic review and authors' preferred surgical technique. Arthroscopy : the journal of arthroscopic & related surgery : official publication of the Arthroscopy Association of North America and the International Arthroscopy Association, 2012. 28(8): p. 1160-9.  Lam, M.T., et al., Patient experience, satisfaction, perception and expectation of osteopathic manipulative treatment: A systematic review. International Journal of Osteopathic Medicine, 2019. 32: p. 28-43.  Li, L., et al., Efficacy and safety of operation versus non-operation for displaced midshaft clavicle fractures: A meta-analysis. Chinese Journal of Evidence-Based Medicine, 2018. 18(5): p. 489-497.  Li, S., et al., Systematic review of patellar resurfacing in total knee arthroplasty. International orthopaedics, 2011. 35(3): p. 305-16.  Lin, G.X., et al., A Systematic Review of Unilateral Biportal Endoscopic Spinal Surgery: Preliminary Clinical Results and Complications. World Neurosurgery, 2019. 125: p. 425-432.  Lin, I.B., et al., Unmet Needs of Aboriginal Australians With Musculoskeletal Pain: A Mixed-Method Systematic Review. Arthritis Care and Research, 2018. 70(9): p. 1335-1347.  Liu, X.Y., et al., What is the optimum fusion technique for adult spondylolisthesis - PLIF or PLF or PLIF plus PLF? A meta-analysis from 17 comparative studies. Spine, 2014. 39(22): p. 1887-1898.  Lohrer, H., S. David, and T. Nauck, Surgical treatment for achilles tendinopathy - A systematic review. BMC Musculoskeletal Disorders, 2016. 17(1).  Losa Iglesias, M.E., et al., Meta-analysis of flexor tendon transfer for the correction of lesser toe deformities. Journal of the American Podiatric Medical Association, 2012. 102(5): p. 359-68.  Louisa, C., et al., Systematic review of consumers' perceived needs of osteoarthritis health information. Internal Medicine Journal, 2016. 46: p. 18-19.  Louw, A., et al., The effect of neuroscience education on pain, disability, anxiety, and stress in chronic musculoskeletal pain. Archives of Physical Medicine and Rehabilitation, 2011. 92(12): p. 2041-2056.  Louw, A., et al., Preoperative education addressing postoperative pain in total joint arthroplasty: review of content and educational delivery methods. Physiotherapy theory and practice, 2013. 29(3): p. 175-94.  Lowe, W.R., et al., Functional Bracing After Anterior Cruciate Ligament Reconstruction: A Systematic Review. The Journal of the American Academy of Orthopaedic Surgeons, 2017. 25(3): p. 239-249.  Ma, Z., et al., Anterior cervical discectomy and fusion versus cervical arthroplasty for the management of cervical spondylosis: a meta-analysis. European Spine Journal, 2017. 26(4): p. 998-1008.  Machotka, Z., et al., Anterior cruciate ligament repair with LARS (ligament advanced reinforcement system): a systematic review. Sports medicine, arthroscopy, rehabilitation, therapy & technology : SMARTT, 2010. 2: p. 29.  Majeed, H., Silastic replacement of the first metatarsophalangeal joint: historical evolution, modern concepts and a systematic review of the literature. EFORT open reviews, 2019. 4(3): p. 77-84.  Makhni, E.C., et al., Outcomes After Shoulder and Elbow Injury in Baseball Players. American Journal of Sports Medicine, 2017. 45(2): p. 495-500.  Malik, A.T., et al., The impact of surgeon volume and hospital volume on postoperative mortality and morbidity after hip fractures: A systematic review. International journal of surgery (London, England), 2018. 54(Pt B): p. 316-327.  Manzoni, A.C.T., et al., The role of the therapeutic alliance on pain relief in musculoskeletal rehabilitation: A systematic review. Physiotherapy Theory and Practice, 2018. 34(12): p. 901-915.  McClellan, C.M., et al., Extended scope physiotherapists in the emergency department: a literature review. Physical Therapy Reviews, 2010. 15(2): p. 106-111.  McDonald, S., S. Hetrick, and S. Green, Pre-operative education for hip or knee replacement. The Cochrane database of systematic reviews, 2004(1): p. Cd003526.  McMahon, S.E., T.O. Smith, and C.B. Hing, A meta-analysis of randomised controlled trials comparing conventional to minimally invasive approaches for repair of an Achilles tendon rupture. Foot and Ankle Surgery, 2011. 17(4): p. 211-217.  Mehta, B.Y., et al., Disparities in outcomes for blacks versus whites undergoing total hip arthroplasty: A systematic literature review. Journal of Rheumatology, 2018. 45(5): p. 717-722.  Morath, O., et al., The effect of sclerotherapy and prolotherapy on chronic painful Achilles tendinopathy—a systematic review including meta-analysis. Scandinavian Journal of Medicine and Science in Sports, 2018. 28(1): p. 4-15.  Muheremu, A., et al., Comparison of the short- and long-term treatment effect of cervical disk replacement and anterior cervical disk fusion: a meta-analysis. European Journal of Orthopaedic Surgery & Traumatology, 2015. 25: p. 87-100.  Nie, H., et al., Comparison of total disc replacement with lumbar fusion: A meta-analysis of randomized controlled trials. Journal of the College of Physicians and Surgeons Pakistan, 2015. 25(1): p. 60-67.  Page, M.J., et al., Patients' experience of shoulder disorders: a systematic review of qualitative studies for the OMERACT Shoulder Core Domain Set. Rheumatology (Oxford, England), 2019.  Palominos, P.E., et al., Fears and beliefs of people living with rheumatoid arthritis: A systematic literature review. Advances in Rheumatology, 2018. 58(1).  Park, Y.H., et al., Implant Arthroplasty versus Arthrodesis for the Treatment of Advanced Hallux Rigidus: A Meta-analysis of Comparative Studies. The Journal of foot and ankle surgery : official publication of the American College of Foot and Ankle Surgeons, 2019. 58(1): p. 137-143.  Parvizi, J., et al., Failure to resurface the patella during total knee arthroplasty may result in more knee pain and secondary surgery. Clinical orthopaedics and related research, 2005. 438: p. 191-6.  Paskins, Z., T. Sanders, and A.B. Hassell, What influences patients with Osteoarthritis to consult their GP about their symptoms? A narrative review. BMC Family Practice, 2013. 14.  Peng, K., et al., Percutaneous endoscopic lumbar discectomy versus conventional discectomy for lumbar disc herniation. International Journal of Clinical and Experimental Medicine, 2016. 9(7): p. 12678-12686.  Phan, K. and R.J. Mobbs, Minimally Invasive Versus Open Laminectomy for Lumbar Stenosis: A Systematic Review and Meta-Analysis. Spine, 2016. 41(2): p. E91-e100.  Phillips, F.M., et al., Lumbar spine fusion for chronic low back pain due to degenerative disc disease: a systematic review. Spine, 2013. 38(7): p. E409-22.  Pierce, T.P., et al., A Systematic Review of Tennis Elbow Surgery: Open Versus Arthroscopic Versus Percutaneous Release of the Common Extensor Origin. Arthroscopy : the journal of arthroscopic & related surgery : official publication of the Arthroscopy Association of North America and the International Arthroscopy Association, 2017. 33(6): p. 1260-1268.e2.  Poder, T.G., et al., A discrete choice experiment on preferences of patients with low back pain about non-surgical treatments: Identification, refinement and selection of attributes and levels. Patient Preference and Adherence, 2019. 13: p. 933-940.  Poh, L.W., et al., An integrative review of experiences of patients with rheumatoid arthritis. International Nursing Review, 2015. 62(2): p. 231-247.  Prasathaporn, N., S. Kuptniratsaikul, and K. Kongrukgreatiyos, Single-row repair versus double-row repair of full-thickness rotator cuff tears. Arthroscopy : the journal of arthroscopic & related surgery : official publication of the Arthroscopy Association of North America and the International Arthroscopy Association, 2011. 27(7): p. 978-85.  Radnay, C.S., et al., Total shoulder replacement compared with humeral head replacement for the treatment of primary glenohumeral osteoarthritis: a systematic review. Journal of shoulder and elbow surgery, 2007. 16(4): p. 396-402.  Roberson, T.A., et al., Outcomes of total shoulder arthroplasty in patients younger than 65 years: a systematic review. Journal of shoulder and elbow surgery, 2017. 26(7): p. 1298-1306.  Roukis, T.S., Percutaneous and minimum incision metatarsal osteotomies: a systematic review. The Journal of foot and ankle surgery : official publication of the American College of Foot and Ankle Surgeons, 2009. 48(3): p. 380-7.  Roukis, T.S., Outcomes after cheilectomy with phalangeal dorsiflexory osteotomy for hallux rigidus: a systematic review. The Journal of foot and ankle surgery : official publication of the American College of Foot and Ankle Surgeons, 2010. 49(5): p. 479-87.  Rushton, A., et al., Physiotherapy rehabilitation post first lumbar discectomy: a systematic review and meta-analysis of randomized controlled trials. Spine, 2011. 36(14): p. E961-72.  Saltzman, B.M., et al., Humeral Head Reconstruction with Osteochondral Allograft Transplantation. Arthroscopy - Journal of Arthroscopic and Related Surgery, 2015. 31(9): p. 1827-1834.  See, M.T.A., et al., Expectations and experiences of patients with osteoarthritis undergoing total joint arthroplasty: An integrative review. International journal of nursing practice, 2018. 24(2): p. e12621.  Shamrock, A., A. Patel, and M. Al Maaieh, The safety profile of percutaneous minimally invasive sacroiliac joint fusion: A systematic review and meta-analysis. Global Spine Journal, 2017. 7(2): p. 43S-44S.  Sharan, D. and J.S. Rajkumar, Physiotherapy for Ankylosing Spondylitis: Systematic Review and a Proposed Rehabilitation Protocol. Current rheumatology reviews, 2017. 13(2): p. 121-125.  Slade, S., et al., What are patient beliefs and perceptions about exercise for non-specific chronic low back pain? A systematic review of qualitative studies. Internal Medicine Journal, 2015. 45: p. 9.  Smith, E. and F.M. Ross, Service user involvement and integrated care pathways. International journal of health care quality assurance, 2007. 20(2-3): p. 195-214.  Swinkels, A., et al., Does patient preference influence the outcomes of exercise in clinical trials of chronic low back pain? Physiotherapy (United Kingdom), 2011. 97: p. eS1196-eS1197.  Taylor, H., et al., CLINICAL, PATIENT-REPORTED OUTCOME MEASURES (PROMS) AND ECONOMIC OUTCOMES ASSOCIATED WITH ENHANCED RECOVERY PROGRAMS IN ELECTIVE HIP AND KNEE SURGERY IN EUROPE, THE MIDDLE EAST AND AFRICA: A SYSTEMATIC LITERATURE REVIEW. Value in Health, 2018. 21: p. S264.  Toye, F., et al., Patients' experiences of chronic non-malignant musculoskeletal pain: a qualitative systematic review. The British journal of general practice : the journal of the Royal College of General Practitioners, 2013. 63(617): p. e829-41.  Turk, D.C. and T.M. Burwinkle, Cognitive-behavioral perspective on chronic pain patients. Critical Reviews in Physical & Rehabilitation Medicine, 2006. 18(1): p. 1-38.  Turner, J.A., et al., Patient Outcomes After Lumbar Spinal Fusions. JAMA: The Journal of the American Medical Association, 1992. 268(7): p. 907-911.  Valdes, K., N. Naughton, and L. Algar, Linking ICF components to outcome measures for orthotic intervention for CMC OA: A systematic review. Journal of hand therapy : official journal of the American Society of Hand Therapists, 2016. 29(4): p. 396-404.  van der List, J.P. and G.S. DiFelice, Role of tear location on outcomes of open primary repair of the anterior cruciate ligament: A systematic review of historical studies. Knee, 2017. 24(5): p. 898-908.  van der Made, A.D., et al., Outcome After Surgical Repair of Proximal Hamstring Avulsions: A Systematic Review. The American journal of sports medicine, 2015. 43(11): p. 2841-51.  Wallis, J.A., et al., Experience of living with knee osteoarthritis: A systematic review of qualitative studies. BMJ Open, 2019. 9(9).  Wiegerinck, J.I., et al., Treatment for insertional Achilles tendinopathy: a systematic review. Knee surgery, sports traumatology, arthroscopy : official journal of the ESSKA, 2013. 21(6): p. 1345-55.  Zaidi, H.A., A.J. Montoure, and C.A. Dickman, Surgical and clinical efficacy of sacroiliac joint fusion: A systematic review of the literature. Journal of Neurosurgery: Spine, 2015. 23(1): p. 59-66.  Zangi, H.A., The evidence for patient education in inflammatory arthritis. Annals of the Rheumatic Diseases, 2014. 73. |
| Wrong study designs or wrong publication methods (n = 21) |
| Abrams, J.S. and J.S. Abrams, Management of the failed rotator cuff surgery: causation and management. Sports Medicine & Arthroscopy Review, 2010. 18(3): p. 188-197.  Anaf, S. and L.A. Sheppard, Physiotherapy as a clinical service in emergency departments: a narrative review. Physiotherapy, 2007. 93(4): p. 243-252.  Anita, E.W., et al., A systematic review of consumer perceived health service needs related to osteoarthritis. Internal Medicine Journal, 2016. 46: p. 18.  Ardern, C.L., J. Kvist, and K.E. Webster, Psychological Aspects of Anterior Cruciate Ligament Injuries. Operative Techniques in Sports Medicine, 2016. 24(1): p. 77-83.  Bitzidis, A. and C. Bitzidou, How painful is osteoporosis? Osteoporosis International, 2017. 28: p. S237-S238.  Brand, C. and S. Cox, Systems for implementing best practice for a chronic disease: Management of osteoarthritis of the hip and knee. Internal Medicine Journal, 2006. 36(3): p. 170-179.  Garcia, G.H., et al., High Satisfaction and Return to Sports after Total Shoulder Arthroplasty in Patients Aged 55 Years and Younger. American Journal of Sports Medicine, 2017. 45(7): p. 1664-1669.  Greig, A., et al., An evaluation of patient-centred care elements that influence patient satisfaction in physiotherapy practice: A systematic review. Physiotherapy (United Kingdom), 2015. 101: p. eS104.  Loughran, I., N. Adams, and N. Caplan, Patient expectation, experience and satisfaction with musculoskeletal physiotherapy. Physiotherapy (United Kingdom), 2017. 103: p. e76-e77.  Louisa, C., et al., Systematic review of consumers' perceived needs of medical services for low back pain. Internal Medicine Journal, 2016. 46: p. 15.  Louisa, C., et al., Systematic review of consumers' perceived needs of healthcare providers for low back pain. Internal Medicine Journal, 2016. 46: p. 15-16.  Louisa, C., et al., Systematic review of consumers' perceived needs of health information for osteoporosis. Internal Medicine Journal, 2016. 46: p. 21.  Louisa, C., et al., Systematic review of consumers' perceived needs of health services for osteoporosis and bone health. Internal Medicine Journal, 2016. 46: p. 21.  Marks, M., et al., Determinants of patient satisfaction after orthopedic interventions to the hand: a review of the literature. Journal of Hand Therapy, 2011. 24(4): p. 303-312.  Papandony, M.C., et al., A systematic review of consumer perceived health service needs related to osteoarthritis. Arthritis and Rheumatology, 2016. 68: p. 1553-1554.  Paskins, Z., T. Sanders, and A.B. Hassell, Comparison of patient experiences of the osteoarthritis consultation with GP attitudes and beliefs to OA: a narrative review. BMC family practice, 2014. 15: p. 46.  Raybould, G., et al., Information needs in patients presenting with a fragility fracture or osteoporosis: A systematic review. Rheumatology (United Kingdom), 2017. 56: p. ii84.  Schoeb, V. and E. Burge, Perceptions of patients and physiotherapists on patient participation: a narrative synthesis of qualitative studies. Physiotherapy research international : the journal for researchers and clinicians in physical therapy, 2012. 17(2): p. 80-91.  Segan, J.D., et al., Systematic review of patient perceived health service needs in inflammatory arthritis. Annals of the Rheumatic Diseases, 2016. 75: p. 1248.  Walters, J.L., S. MacKintosh, and L. Sheppard, The journey to total hip or knee replacement. Australian Health Review, 2012. 36(2): p. 130-135.  Yung, V., et al., Patient satisfaction with musculoskeletal physiotherapy care in Australia is high. Physiotherapy (United Kingdom), 2011. 97: p. eS1376-eS1377. |
| Not musculoskeletal disorders or not adult population (n = 16) |
| Adkinson, J., et al., Do Patient- and Parent-reported Outcomes Measures for Children With Congenital Hand Differences Capture WHO-ICF Domains? Clinical Orthopaedics & Related Research, 2015. 473(11): p. 3549-3563.  Adler, R., A. Vasiliadis, and N. Bickell, The relationship between continuity and patient satisfaction: a systematic review. Family practice, 2010. 27(2): p. 171-8.  Alanko, O.M., A.L. Svedstrom-Oristo, and M.T. Tuomisto, Patients' perceptions of orthognathic treatment, well-being, and psychological or psychiatric status: a systematic review. Acta odontologica Scandinavica, 2010. 68(5): p. 249-60.  Armas, A., et al., Face-to-face communication between patients and family physicians in Canada: A scoping review. Patient education and counseling, 2018. 101(5): p. 789-803.  Aslakson, R.A., J.R. Curtis, and J.E. Nelson, The changing role of palliative care in the ICU. Critical care medicine, 2014. 42(11): p. 2418-28.  Beck, R.S., R. Daughtridge, and P.D. Sloane, Physician-patient communication in the primary care office: a systematic review. The Journal of the American Board of Family Practice, 2002. 15(1): p. 25-38.  Boss, E.F., et al., Shared Decision Making and Choice for Elective Surgical Care: A Systematic Review. Otolaryngology-Head & Neck Surgery, 2016. 154(3): p. 405-420.  Fradgley, E.A., C.L. Paul, and J. Bryant, A systematic review of barriers to optimal outpatient specialist services for individuals with prevalent chronic diseases: what are the unique and common barriers experienced by patients in high income countries? International journal for equity in health, 2015. 14: p. 52.  Frendl, D.M., M. Strom, and J.E. Ware Jr, Patient reported health outcomes from well-controlled trials of biologic therapies: A systematic review. Pharmacoepidemiology and Drug Safety, 2013. 22: p. 436-437.  Jiang, D., W. Kong, and J.J. Jiang, Patient engagement in randomized controlled tai chi clinical trials among the chronically ill. Reviews on Recent Clinical Trials, 2016. 11(4): p. 1-10.  Kierkegaard, S., et al., Time course of patient reported outcomes after hip arthroscopic surgery for femoroacetabular impingement-a systematic review with meta-analysis. Osteoarthritis and Cartilage, 2016. 24: p. S513-S514.  Lang, S., M. Velasco Garrido, and C. Heintze, Patients' views of adverse events in primary and ambulatory care: a systematic review to assess methods and the content of what patients consider to be adverse events. BMC Family Practice, 2016. 17: p. 1-9.  Oliveira, V., et al., The effect of patient-health provider communication on satisfaction with care: A systematic review. Physiotherapy (United Kingdom), 2011. 97: p. eS936-eS937.  Pinto, R.Z., et al., Which verbal and nonverbal communication behaviours influence the therapeutic alliance between patients and health practitioners? systematic review of the literature. Physiotherapy (United Kingdom), 2011. 97: p. eS1004-eS1005.  van Deventer, C. and P. McInerney, Patients' involvement in their own care through quality improvement initiatives: A systematic review of qualitative and opinion evidence. JBI Database of Systematic Reviews and Implementation Reports, 2012. 10(57): p. 3936-3948.  Whear, R., et al., Patient initiated clinics for patients with chronic or recurrent conditions managed in secondary care: a systematic review of patient reported outcomes and patient and clinician satisfaction. BMC health services research, 2013. 13: p. 501. |
| Language not in inclusion criteria (n = 3) |
| Duivenvoorden, T., et al., [Patient expectations and satisfaction concerning total knee arthroplasty]. Nederlands tijdschrift voor geneeskunde, 2017. 160: p. D534.  Schulze, A. and H.P. Scharf, [Satisfaction after total knee arthroplasty. Comparison of 1990-1999 with 2000-2012]. Der Orthopade, 2013. 42(10): p. 858-65.  Buchholz, I. and T. Kohlmann, [Patient goals for medical rehabilitation - overview of the current state of research in Germany]. Die Rehabilitation, 2013. 52(2): p. 75-85. |
